# RNS60 exerts therapeutic effects in the SOD1 ALS mouse model through protective glia and peripheral nerve rescue

**DOI:** 10.1186/s12974-018-1101-0

**Published:** 2018-03-01

**Authors:** Antonio Vallarola, Francesca Sironi, Massimo Tortarolo, Noemi Gatto, Roberta De Gioia, Laura Pasetto, Massimiliano De Paola, Alessandro Mariani, Supurna Ghosh, Richard Watson, Andreas Kalmes, Valentina Bonetto, Caterina Bendotti

**Affiliations:** 1 Molecular Neurobiology Lab, Department of Neuroscience, IRCCS - Mario Negri Institute, Via La Masa, 19, 20156 Milan, Italy; 2 Translational Biomarkers Lab, Department of Molecular Biochemistry and Pharmacology, IRCCS - Mario Negri, Milan, Italy; 3 Analytical Biochemistry Lab, Department of Environmental Health Sciences, IRCCS- Mario Negri Institute, Milan, Italy; 4 Revalesio Corporation, Tacoma, Washington, USA

**Keywords:** ALS, RNS60, Neuroinflammation, Oxidative stress

## Abstract

**Background:**

Amyotrophic lateral sclerosis (ALS) is a progressive neurodegenerative disease that affects the motor neuromuscular system leading to complete paralysis and premature death. The multifactorial nature of ALS that involves both cell-autonomous and non-cell-autonomous processes contributes to the lack of effective therapies, usually targeted to a single pathogenic mechanism. RNS60, an experimental drug containing oxygenated nanobubbles generated by modified Taylor-Couette-Poiseuille flow with elevated oxygen pressure, has shown anti-inflammatory and neuroprotective properties in different experimental paradigms. Since RNS60 interferes with multiple cellular mechanisms known to be involved in ALS pathology, we evaluated its effect in in vitro and in vivo models of ALS.

**Methods:**

Co-cultures of primary microglia/spinal neurons exposed to LPS and astrocytes/spinal neurons from SOD1^G93A^ mice were used to examine the effect of RNS60 or normal saline (NS) on the selective motor neuron degeneration. Transgenic SOD1^G93A^ mice were treated with RNS60 or NS (300 μl/mouse intraperitoneally every other day) starting at the disease onset and examined for disease progression as well as pathological and biochemical alterations.

**Results:**

RNS60 protected motor neurons in in vitro paradigms and slowed the disease progression of C57BL/6-SOD1^G93A^ mice through a significant protection of spinal motor neurons and neuromuscular junctions. This was mediated by the (i) activation of an antioxidant response and generation of an anti-inflammatory environment in the spinal cord; (ii) activation of the PI3K-Akt pro-survival pathway in the spinal cord and sciatic nerves; (iii) reduced demyelination of the sciatic nerves; and (iv) elevation of peripheral CD4+/Foxp3+ T regulatory cell numbers. RNS60 did not show the same effects in 129Sv-SOD1^G93A^ mice, which are unable to activate a protective immune response.

**Conclusion:**

RNS60 demonstrated significant therapeutic efficacy in C57BL/6-SOD1^G93A^ mice by virtue of its effects on multiple disease mechanisms in motor neurons, glial cells, and peripheral immune cells. These findings, together with the excellent clinical safety profile, make RNS60 a promising candidate for ALS therapy and support further studies to unravel its molecular mechanism of action. In addition, the differences in efficacy of RNS60 in SOD1^G93A^ mice of different strains may be relevant for identifying potential markers to predict efficacy in clinical trials.

**Electronic supplementary material:**

The online version of this article (10.1186/s12974-018-1101-0) contains supplementary material, which is available to authorized users.

## Background

Amyotrophic lateral sclerosis (ALS) is a fatal adult-onset neurodegenerative disorder that affects the motor neuromuscular system leading to progressive muscle weakness, paralysis and death by respiratory failure within 3–5 years after diagnosis [1, 2]. Until now, only two therapeutic agents, riluzole and edaravone, developed as potential antiglutamatergic and free radical scavenger respectively, have been approved by the Food and Drug Administration (FDA) for treatments aimed at slowing ALS disease progression. However, while riluzole marginally extends patient life by 2–3 months [3, 4], edaravone so far has only shown a slowing of the rate of decline of the ALSFRS-R score compared with placebo in a small subset of ALS patients at early disease stage [5]. This paucity of therapies is in part due to the multifactorial nature of ALS pathogenesis that involves both cell-autonomous and non-cell-autonomous processes [6]. The cause of ALS remains unknown except for the genetic forms that represent only about 15–20% of all cases and are clinically indistinguishable from the sporadic form of ALS. The affected genes are involved in various cellular functions, suggesting a large variety of possible disease mechanisms. Although more than 20 different genes have been linked to ALS [7], to date, the most reliable and informative disease model is the transgenic mouse overexpressing human superoxide dismutase 1 (hSOD1), which recapitulates the main pathological traits of ALS [8]. Evidence from these mice and from ALS patients indicates that multiple mechanisms are involved in the death of the motor neurons (MNs) and the loss of muscular innervations. They include dysfunctions intrinsic to motor neurons such as protein aggregation, impaired RNA processing and metabolism, mitochondrial dysfunction, oxidative stress, cytoskeletal alterations and impairment of axonal transport [6, 9]. Other extrinsic mechanisms involve additional cell types of the central nervous system (CNS) (such as reactive astroglia and microglia) or the peripheral immune system (like macrophages and T-cells) that may elicit neuroinflammatory processes contributing actively to motor neuron degeneration [10–12]. Motor neurons become vulnerable to these mechanisms in adult life, through the loss of compensatory pro-survival mechanisms, creating a disbalance between the signaling pathways of survival and death. Protective compensatory mechanisms may include the activation of pro-survival pathways such as the phosphatidylinositol-3 kinase (PI3K)-Akt pathway [13] and/or the induction of an anti-inflammatory environment [14]. It has been reported that neurotrophic factors, e.g., IGF1, GDNF and VEGF [15–17], are able to rescue motor neurons in SOD1 mutant mice through activation of the p-Akt pathway. On the other hand, a newly emerging hypothesis states that adaptive immunity through Th2 cells or and T regulatory cells (Treg cells) may initially activate the protective function of microglia or macrophages in the spinal cord, thereby providing a protective milieu to sustain motor neuron viability. As the disease progresses, the decline in Treg and the increase of Th1-mediated immunity lead to a shift of the microglia/macrophages toward a more proinflammmatory phenotype, which accelerates motor neuron degeneration [18]. Consistent with this hypothesis, we recently demonstrated that in C57BL/6-SOD1^G93A^ mice there was a prominent immune response in the motor neurons and sciatic nerves compared to a variant of the model in a different genetic background (129Sv-SOD1^G93A^ mice), and this was associated with a slower disease progression rate, preservation of myelin sheath morphology and a delayed neuromuscular junction (NMJ) denervation in the C57BL/6 model [19–21]. Therefore, sustaining the initial compensatory mechanisms, in addition to inhibiting the toxic mechanisms, is likely to be the best strategy to significantly slow down the disease progression in ALS.

RNS60 is a novel experimental drug containing oxygen-filled charge-stabilized nanostructures (CSNs) generated using modified Taylor-Couette-Poiseuille (TCP) flow under elevated oxygen pressure [22]. CSNs consist of an oxygen core surrounded by layers of positive and negative electrical charges [23]. The biological effects of RNS60 have been attributed to the physical properties of the CSNs, as a control solution containing a similar level of oxygen as RNS60 (50–60 ppm) and not subjected to TCP flow modification is not biologically effective [22]. The putative mechanisms of action of RNS60 encompass activation of pro-survival pathways, increase in mitochondrial biogenesis, protection of myelin forming cells, reduction of inflammation, and increase of Tregs [22, 24]. For example, RNS60 attenuates the expression of pro-inflammatory molecules in activated mouse microglia cells through the activation of the PI3-kinase-Akt pathway [22]. Additional studies have shown that RNS60 protects dopaminergic neurons and improves motor function in a mouse model of Parkinson disease by activating the pro-survival PI3K-Akt pathway and reducing microglial activation [23]. Moreover, it showed improvement of learning and memory in a transgenic model of Alzheimer disease by protecting hippocampal and cortical neurons, preventing tau phosphorylation and upregulating neuronal plasticity [25, 26]. Another positive effect of RNS60 is its ability to increase synaptic transmission by upregulating ATP synthesis in mitochondria [27, 28] and decrease fatigability of nerve stimulated muscles [29]. Finally, it is able to inhibit disease progression in multiple sclerosis rodent models by regulating the immune responses and presumably by promoting myelin maintenance and repair [24, 30–32].

Therefore, RNS60 seems to influence multiple cellular mechanisms that are also involved in ALS pathology suggesting that it may have a potential beneficial impact in this disease as well. Accordingly, in the present study we investigated the effect of RNS60 in both in vitro and in vivo models of familial ALS linked to SOD1^G93A^ mutation.

## Methods

### Preparation of RNS60

RNS60 was generated at Revalesio (Tacoma, WA) using a rotor/stator device that incorporates controlled turbulence and Taylor-Couette-Poiseuille flow as described before [22]. The resulting fluid was immediately placed into glass bottles (KG-33 borosilicate glass, Kimble-Chase) and sealed using gray chlorobutyl rubber stoppers (USP class 6, West Pharmaceuticals) to maintain pressure and minimize leachables. When tested after 24 h, the oxygen content was 55 ± 5 ppm. Chemically, RNS60 only contains water, sodium chloride, and 50–60 ppm oxygen [22]. Normal saline (NS) from the same manufacturing batch was used as control. For the first in vitro experiment on motor neuron toxicity induced by LPS-activated microglia, we used an additional control ONS60, which is saline with a similar oxygen content as RNS60 (55 ± 5 ppm) but not processed with TCP flow. ONS60 contacted the same device surfaces as RNS60 and was bottled in the same way.

### SOD1^G93A^ mice

Transgenic animals (B6SJL-TgN SOD-1-SOD1.G93A-1Gur) were originally obtained from Jackson laboratories (USA) and then maintained on a C57BL6/J or 129Sv background (following indicated as C57BL/6-SOD1^G93A^ or 129Sv**-**SOD1^G93A^, respectively) at the Mario Negri Institute for Pharmacological Research, Milan, Italy (IRFMN). The animals were housed under SPF (specific pathogen free) standard conditions (22 ± 1 °C, 55 ± 10% relative humidity and 12-h light/dark schedule), 3–4 per cage, with free access to food (standard pellet, Altromin, MT, Rieper) and water. Procedures involving animals and their care were conducted in conformity with the institutional guidelines of the Mario Negri Institute for Pharmacological Research, Milan, Italy IRFMN, which are in compliance with national (D.lgs 26/2014; Authorization n.19/2008-A issued March 6, 2008 by Ministry of Health) and Mario Negri Institutional regulations and Policies providing internal authorization for persons conducting animal experiments (Quality Management System certificate—UNI EN ISO 9001:2008—reg. No. 6121); the NIH Guide for the Care and Use of Laboratory Animals (2011 edition) and EU directives and guidelines (EEC Council Directive 2010/63/UE). The Statement of Compliance (Assurance) with the Public Health Service (PHS) Policy on Human Care and Use of Laboratory Animals has been newly reviewed (9/9/2014) and will expire on September 30, 2019 (animal Welfare Assurance #A5023-01).

### Primary microglia-motor neuron co-cultures

Primary cells for the microglia/motor neuron co-cultures were obtained from the spinal cord of 13-day-old C57BL/6J mouse embryos as previously reported [33]. The viability of motor neurons was assayed as previously described [33]. Only the SMI32-positive cells, with typical morphology triangular shape, single well-defined axon, large bodies diameter ≥ 20 μm) and intact axons and dendrites were counted; this number was normalized to the mean of SMI32-positive cells counted in the appropriate control wells. After 6 days in vitro were exposed to LPS for 24 h to induce the motor neuron death. RNS60 10% *v*/*v* or NS were added 2 h before the LPS.

### Primary astrocyte-spinal neuron co-cultures

Primary SOD1^G93A^ and non-transgenic (NTG) astrocytes were prepared as previously reported [13, 34, 35]. Briefly, cortices of E13-E14 embryos from C57BL/6-SOD1^G93A^ mice or their NTG littermates were dissected and mechanically dissociated in Hanks’ balanced salt solution (HBSS) containing 33 mM glucose. After centrifugation, the pellet was resuspended in culture medium (Dulbecco’s modified Eagle’s medium/F12, 2 mM l-glutamine, 33 mM glucose, 5 lg/mL gentamycin, 10% horse serum] and seeded (500,000 cells/mL) onto 48- or 6-well plates coated with 1.5 μg/mL poly-l-ornithine. Repeated washing with HBSS, 12 h of orbital shaking at 200 rpm and treatment with 10 M AraC once they reached confluence, rendered astrocyte cultures free of microglia, oligodendrocytes, and neurons. SOD1^G93A^ and non-transgenic co-cultures were prepared as previously described [13, 35]. Spinal cords of E13-E14 embryos were dissected and mechanically dissociated in HBSS, 33 mM glucose. The cells were centrifuged onto a 4% bovine serum albumin cushion at 1000 rpm for 10 min and the pellet resuspended in neuron culture medium: Neurobasal (Gibco, Rockville, MD, USA), 2 mM l-glutamine, 33 mM glucose, 5 μg/mL gentamycin, 1 ng/mL brain-derived neurotrophic factor, 25 μg/mL insulin, 10 μg/mL putrescine, 30 nM sodium selenite, 2 μM progesterone, 100 μg/mL apo-transferrin, 10% heat-inactivated horse serum, and 10 uM AraC. Cells were seeded (1,000,000 cells/ml) onto 48-well plates with a pre-established astrocyte confluent layer to obtain spinal neuron-cortical astrocyte co-cultures. Non-transgenic and SOD1^G93A^ co-culture were obtained from non-transgenic and SOD1^G93A^ neurons seeded on non-transgenic and SOD1^G93A^ astrocytes, respectively. Motor neurons obtained from E13-14 embryos are fully differentiated and express the specific transcription factors Hb9 and Islet-1/2. Few days after plating, they show adult characteristics such as profuse dendrite and axon outgrowth and synapse formation [36–38]. Co-cultures were treated twice (DIV 0 and 3) with 10% *v*/*v* of RNS60 or normal saline (NS) or oxygenated normal saline (ONS) as controls and fixed at DIV 6.

### Immunocytochemistry and evaluation of motor neuron survival in astrocyte-spinal neuron co-cultures

Immunocytochemistry was run as previously described [35, 39]. After blocking the non-specific binding sites by incubation with a solution containing 10% normal goat serum (NGS) and Triton in PBS (phosphate-buffered saline) 0.01 M, the cells were incubated with the primary antibodies (overnight at 4 °C), diluted in a solution containing 1% NGS and Triton in PBS 0.01 M. We used the following primary antibodies: anti-SMI32 (1:1000, Covance, Princeton, NJ, USA) and anti-NeuN (1:250, Chemicon, Temecula, CA, USA). Cells were then washed and incubated with the appropriate secondary fluorescent antibody (1:500, Alexa Fluor Dyes, Life Technologies, Grand Island, NY, USA) or secondary biotinylated antibody (1:500, Vector Laboratories, Burlingame, CA, USA) for tyramide signal amplification (TSA, Perkin Elmer) following the manufacturer’s instructions. Images were acquired with Olympus BX41 fluorescence microscope. Motor neuron survival was evaluated as previously described [35, 39]. The labeling with anti-SMI32 antibody highlights motor neurons with typical morphology and large cell bodies (diameter ≥ 20 μm) and anti-NeuN antibody was used to identify all neurons in twelve adjacent frames per well at × 10 magnification. Data were expressed as the ratio of the number of motor neurons to the total neurons.

### Neurite outgrowth evaluation

For morphological analysis, we used Image J software (http://imagej.nih.gov/ij/) with the NeuronJ plug in [40]. Cells were visualized by SMI32 staining and analyzed for the following parameters: total neurites extensions per neuron and number of neurites per neuron. Neurites were manually traced and measured on size-calibrated images starting from the soma of motor neurons, 5 independent experiments with each *N* = 15 motor neurons each were analyzed per condition.

### RNS60 mouse treatments

Female C57BL/6-SOD1^G93A^ or 129Sv-SOD1^G93A^ mice were treated every other day by intraperitoneal (i.p.) injection with 300 μl of RNS60 or Normal Saline (NS). Treatments of SOD1^G93A^ mice (22 mice per group) started from the onset of the disease evaluated when the body weight is at the peak level and the hind limbs show first signs of tremors and reduced abduction but not impairment in motor performance and muscle strength. This occurs at 15 weeks of age in our mouse colony; therefore, all mice start the treatment at 105 days. A set of mice (8 per group) were sacrificed at 20 weeks of age (symptomatic stage) for histopathological, biochemical and molecular analysis. The rest of each group (*n* = 14) were followed until the end stage to assess the effect on survival. Treatment of 129Sv-SOD1^G93A^ mice (10 mice per group) started from the 13th week of age (onset of the disease assessed as above) until 16 weeks of age (symptomatic stage) when they were sacrificed for histopathological, biochemical and molecular analysis.

### Behavioral analysis, motor dysfunction assessment and survival

Evaluation of disease was performed in all mice twice a week from the onset of the disease, by the same investigator blinded to the treatment. The grip strength test was used to measure the limb resistance as previously described [35]. Mice were placed on a horizontal metallic grid which was then gently inverted. The latency to fall of each mouse was recorded. The test ended after 90 s. In the case of failure, the measurement was repeated three times and the best performance of the session was considered for the statistical analysis. We evaluated the onset of neuromuscular symptoms at the age when the mouse exhibits the first failure in the paw grip strength test at two consecutive time points. The age at which the mice were no longer able to perform the grip strength test was considered as time of paralysis. The mice were euthanized by deep anesthesia when they were unable to right themselves within 10 s after being placed on either side. This time was considered the end stage of the disease and was used to calculate the survival interval from onset.

### Immunohistochemistry

Spinal cords were processed as previously described [35]. Briefly, under deep anesthesia, mice were transcardially perfused with PBS followed by 4% paraformaldehyde (PFA) solution. The spinal cords were quickly removed, postfixed for 24 h, and cryopreserved in 30% sucrose solution at 4 °C until they sank, included in Tissue-tek OCT (Sakura, Zoeterwounde, The Netherlands), frozen in n-pentane at − 45 °C and stored at − 80 °C until analysis. Spinal cord immunohistochemistry was performed on free floating sections (30 μm). The number of motor neurons was determined on serial sections (20, one every ten) from lumbar spinal cord segments L2-L5 of 3 mice per group as previously described [13]. The sections were stained with cresyl violet to detect the Nissl substance of neuronal cells. Motor neuron counting was performed at × 10 magnification using the free software ImageJ (http://imagej.nih.gov/ij/), previously calibrated. NISSL labeled neurons with clear nucleus and nucleolus and an area of the cell body higher than 400 μm^2^ was identified as motor neurons. The number of motor neurons was calculated for each hemisection and the means used for statistical analysis. Immunofluorescence was evaluated on 5–7 coronal spinal cord slices (one every ten) from lumbar spinal cord per animal. The following primary antibodies and staining methods were used: mouse anti-SMI-31 (1:1000, Sternberger Inc); rabbit anti-Iba1 (1:1000, Wako); mouse anti-GFAP (1:2500, Millipore); rat anti-CD68 (1:200, AbDSerotec); rabbit anti-hSOD1 (1:1500, Upstate); rabbit anti-S100β (1:3000, Sigma), and neurotrace conjugated with Alexa-647 (1:500, Invitrogen). Appropriate fluoro-conjugated secondary antibodies (1:500 dilution) were used: Alexa 549, Alexa 647 and Alexa 488 (Alexa Fluor® Dyes, Life Technologies). The sections were analyzed under Olympus Fluoview confocal microscope. The quantification of GFAP, CD68, IBA1, and S100β intensity was carried out, by determining the mean gray value of fluorescent signal in the ventral horns in the relative surface occupied by the staining. The quantification of SMI31 positive cell was evaluated considering the cells double stained with SMI31 and neurotrace with a typical motor neuronal morphology. The quantification of hSOD1 signal was evaluated by determining the area fraction of fluorescent signal in the ventral horns of lumbar spinal cord. All these analyses were carried out using the free software ImageJ (http://imagej.nih.gov/ij/) by the same operator blinded to treatment, by determining a threshold value within a gray-scale (corresponding to the maximum level of an unstained section background) and considering as positive events all the pixels falling over this value. The cellular distribution of p-Akt was assessed by immunohistochemistry using the tyramide amplification protocol as previously described [39]. Briefly, the endogenous peroxidases were inactivated by 1% hydrogen peroxide in TBS (0.1 M Tris-HCl, 0.14 M NaCl, pH 7.4). The sections were then incubated with 3% bovine serum albumin (BSA) in TBS/Triton 0.1% 1 h at RT, and then probed overnight at 4 °C in 3% BSA, TBS/Triton with an anti-p-Akt rabbit polyclonal antibody, 1:500 dilution, from Cell Signaling. Subsequently, the sections were washed in TBS/Triton and incubated 1 h at RT in 1% BSA, TBS with a secondary biotinylated anti-rabbit antibody, 1:200 dilution, from Vector. The secondary antibody was revealed with a TSA amplification kit, Cy5 (Perkin Elmer). The sections were then immunostained for GFAP according to the protocols described above. For each immunofluorescence procedure, some of the sections were processed without primary antibody, to verify the specificity of the staining.

### Neuromuscular junctions (NMJs)

For detection of the NMJ denervation according to the previously described protocol [35], tibialis anterior muscles (TAM) were dissected and snap-frozen in isopentane cooled in liquid nitrogen. Cryostat sections of 20 μm collected on poli-lysine objective slides (VWR International) were fixed in chilled acetone for 10 min, incubated in a blocking solution (0.3% Triton, 10% NGS in 0.01 M PBS) for 1 h at 22 °C and left overnight at 4 °C with anti-synaptophysin primary antibody (rabbit, 1:100, Synaptic System) in 0.15% Triton, 5% NGS, 0.01 M PBS. Then the sections were incubated with goat anti-rabbit 647 (1:500, Alexa Fluor® Dyes, Life Technologies) secondary antibody and with bungarotoxin (1:500, Invitrogen) conjugated with Alexa Fluor® 594 (Life Technologies). Innervated neuromuscular junctions were identified by labeling with bungarotoxin, totally or partially co-localized with synaptophysin. Plaques marked with bungarotoxin only were considered denervated and were expressed as the percentage of the total plaques (counted in 8 adjacent frames per section). Five sections, at  20X magnification, were analyzed for each mouse. Tibialis anterior muscle was also used to analyze the infiltration of CD68^+^ cells in the vicinity of the NMJ using an antibody rat anti-CD68 (1:200, AbD Serotec) after the incubation in a blocking solution (0.1% Triton, 10% NGS in 0.01 M PBS) for 1 h at 22 °C and left overnight at 4 °C with the primary antibody in 0.1% Triton, 3% NGS, 0.01 M PBS. Fluorescence images along the *z*-axis were taken by Olympus confocal microscopy using a  20X objective and z-stacking was performed by using ImageJ/Fiji software (with Z-StackProjection/SUM command; National Institutes of Health).

### Western blot

Mice were sacrificed according to institutional ethical procedures by decapitation, and the spinal cord and sciatic nerve were rapidly dissected. The spinal cord was immediately frozen on dry ice and stored at − 80 °C. For each mouse, the lumbar spinal cord was longitudinally transected at 50 μm in a cryostat with ventral and dorsal spinal cord sections as separate samples. The resulting cryostat ventral material was homogenized by sonication in ice-cold homogenization buffer (20 mM Tris-HCl pH 7.4, 2% Triton X-100, 150 mM NaCl, 1 mM EDTA, 5 mM MgCl_2_, anhydrous glycerol 10%, protease and phosphates inhibitor cocktail, Roche), centrifuged at 13000 rpm for 30 min at 4 °C, and the supernatants were collected and stored at − 80 °C. The sciatic nerves were processed as previously described [19]. Briefly, tissues were powdered in liquid nitrogen, next homogenized by sonication in ice-cold homogenization buffer (20 mM Tris-HCl pH 7.4, 2% Triton X-100 1%, 150 mM NaCl, 1 mM EDTA, 5 mM MgCl_2_, anhydrous glycerol 10%, protease and phosphates inhibitor cocktail, Roche) and centrifuged at 13,000 rpm for 15 min at 4 °C. Equal amounts of total protein homogenates were loaded on polyacrylamide gels and electroblotted onto PVDF membrane (Millipore) as previously described [35]. Blots were first blocked with 5% BSA (Sigma) in TBS/Tween 0.1% for 1 h at room temperature and then over night at 4 °C with one of the following primary antibodies: rabbit monoclonal anti-p-Akt (1:750, Cell Signaling), anti-Akt, (1:1000, Cell Signaling), anti-p-GSK3β (1:1000 rabbit polyclonal specific for phospho-Ser9 of GSK3β, Cell Signaling), rat anti-MBP (1:1000 Chemicon), mouse anti CNPase (1:1000 Chemicon), rabbit polyclonal anti-Nrf2 (1:200, Santa Cruz Biotechnology), mouse monoclonal anti-GFAP (1:1000 millipore), mouse monoclonal anti β-actin (1:15000 Chemicon), and mouse monoclonal anti-GAPDH (1:20000, Millipore). Membranes were then washed and incubated with horseradish peroxidase-conjugated anti-rabbit, anti-mouse or anti-rat secondary antibodies (Santa Cruz) and developed by Luminata Forte Western Chemiluminescent horse radish peroxidase (HRP) Substrate (Millipore) on the Chemi-Doc XRS system (Bio-Rad). Densitometry analysis was performed with Quantity One (Bio-Rad) software.

### Dot blot analysis

Proteins (3 μg) were directly loaded onto nitrocellulose Trans-Blot transfer 0.45 μm (Bio-Rad) membranes, depositing each sample on the membrane by vacuum filtration on a Bio-Dot Microfiltration Apparatus (Bio-Rad), as described previously [41]. Dot blot membranes were blocked with 3% (*w*/*v*) BSA (Sigma) and 0.1% (*v*/*v*) Tween 20 in Tris-buffered saline, pH 7.5, incubated with mouse monoclonal anti-NT (1:1000; Merck-Millipore), then with anti-mouse peroxidase-conjugated secondary antibody (Santa Cruz Biotechnology Inc.). Blots were developed with Luminata™ Forte Western Chemiluminescent HRP Substrate (Millipore) on the ChemiDoc XRS system (Bio-Rad). Densitometry was done with Progenesis PG240 v2006 software (Nonlinear Dynamics). NT immunoreactivity was normalized to the actual amount of proteins loaded on each dot in the membrane as detected after Ponceau Red staining (Fluka). Values were expressed as mean ± SEM.

### Real-time PCR

Tissues (spinal cords and spleen) were freshly collected and immediately frozen on dry-ice. The ventral portion of lumbar spinal cord and a segment of spleen was homogenized, and total RNA from spinal cord was extracted using the Trizol method (Invitrogen) and purified with PureLink RNA columns (Life Technologies). RNA samples were treated with DNase I and reverse transcription was performed with High Capacity cDNA Reverse Transcription Kit (Life Technologies). Real-time PCR was performed using the Taq Man Gene expression assay (Applied Biosystems) following the manufacturer’s instructions, on cDNA specimens in triplicate, using 1x Universal PCR master mix (Life technologies) and 1x mix containing specific receptors probes for the interleukin 1 beta (IL1β Mm00434228_m1), chemokine (C-C motif) ligand 2 (CCL2, Mm00441242_m1), interleukin 4 (IL-4, Mm00445259_m1), chitinase-like 3 (Ym1, Mm00657889_mH), forkhead box P3 (FoxP3, Mm00475162_m1), and cluster of differentiation 4 (CD4, Mm00442754_m1) (Life Technologies). Relative quantification was calculated from the ratio between the cycle number (Ct) at which the signal crossed a threshold set within the logarithmic phase of the given gene and that of the reference β-actin gene (4310881E; Life technologies). Mean values of the triplicate results for each animal were used as individual data for 2^−ΔΔCt^ statistical analysis.

### Statistical analysis

One-way or two-way ANOVA was used to compare differences between more than two groups, post hoc Fisher’s least significant difference (LSD). Two-way ANOVA for repeated measures was used to compare the behavioral analysis. The Mantel-Cox log rank test was used for comparing motor deficit onset, paralysis and survival between groups.

## Results

### RNS60 protects motor neurons in two different cellular models of ALS

**Fig. 1:**
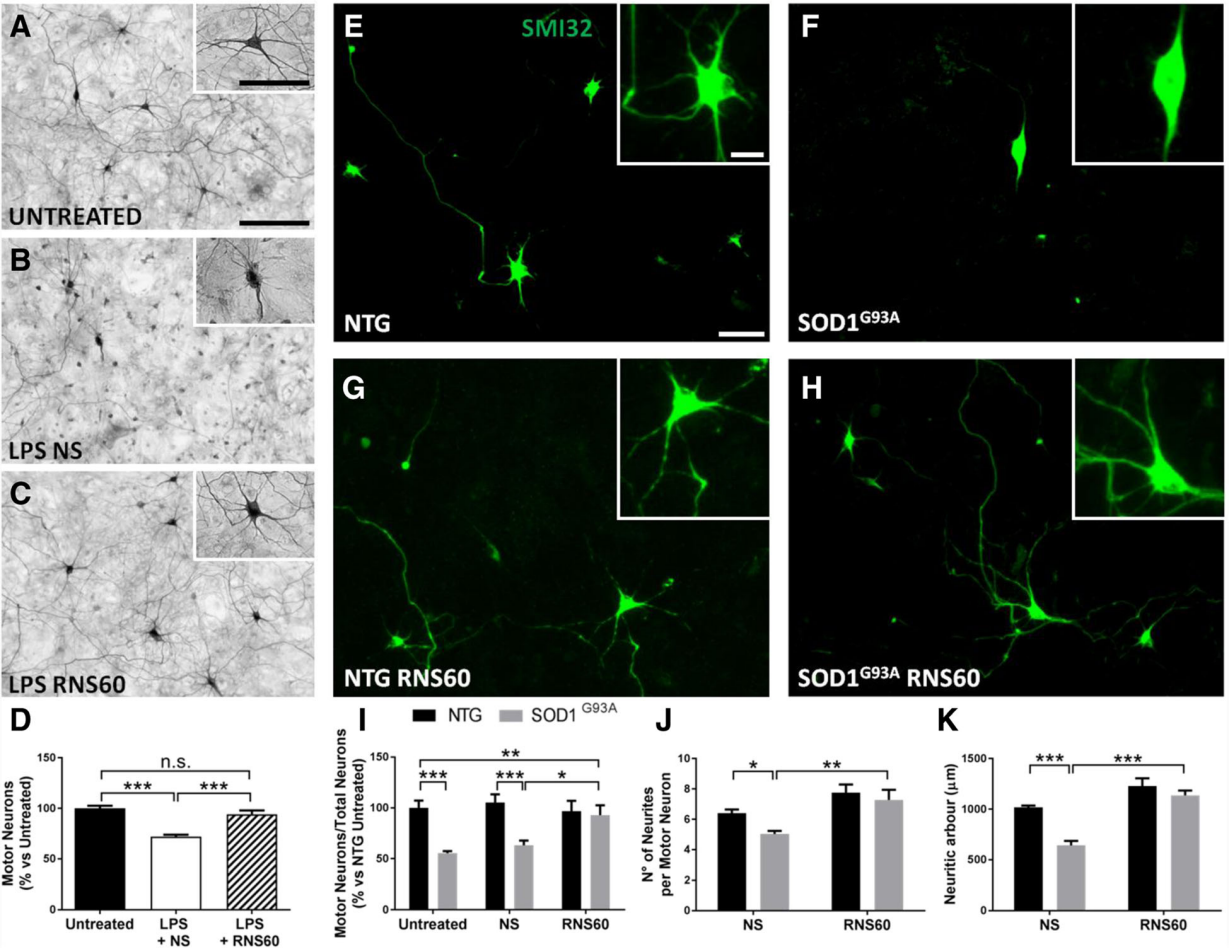
RNS60 protects MNs from death in two cellular models of ALS. **a**–**c** Representative images of primary microglia-MN enriched co-cultures exposed to LPS for 24 h after 6 DIV. **e**–**h** MNs were identified by morphology, intense SMI32 immunolabeling and diameter > 20 μm. **d** The bar graph indicates that LPS reduces the viability of control (NS) treated MNs by about 30%. This toxic effect was significantly prevented by RNS60 (10% *v*/*v*). Data are expressed as mean ± SEM (*n* = 6), One-way ANOVA (*p* < 0.001) followed by post hoc Fisher’s LSD. ***p < 0.001. **e**–**h** Representative images of SMI32-(green) labeled MNs in NTG and C57BL/6-SOD1^G93A^ co-cultures treated with NS or RNS60 (scale bar: 50 μm). Inserts show MNs with the neuritic arbor at higher magnification (scale bar: 20 μm). **i** Quantitative assessment of MN survival in astrocyte-spinal neuron co-cultures from NTG (black) or SOD1^G93A^ (gray) co-cultures. Cells were treated with 10% *v*/*v* of RNS60 or NS, or left untreated as control. The columns show the number of viable MNs (as a percentage of NTG untreated samples). Data are expressed as the ratio between the MN number (SMI32-positive, maximum diameter > 20 μm) and the number of total NeuN-positive neurons. Only treatment with RNS60 completely prevented MN loss in transgenic co-cultures. Data are expressed as mean ± SEM (*n* = 5 independent experiment), Two-way ANOVA (*p* < 0.04) followed by post hoc Fisher’s LSD. **j**, **k** The analysis of the neuritic outgrowth of the MNs revealed a reduction of number and total extensions of neurites in transgenic co-cultures. RNS60 was able to significantly prevent the decrease of both parameters. Data are mean ± SEM (*n* = 5). Data were analyzed with two-way ANOVA followed by post hoc Fisher’s LSD: *p < 0.05, **p < 0.01, ***p < 0.001, n.s. = non-significant

The effect of RNS60 was tested initially on motor neuron/glia co-cultures derived from C57BL/6J mouse embryos. The co-cultures were exposed to 1 μg/mL lipopolysaccharide (LPS) for 24 h after 6 days in vitro (DIV). In this paradigm, co-treatment with LPS and 10% *v*/*v* of normal saline (NS) reduced MN viability by about 30% (72.12 ± 1.82% vs 100 ± 2.51% in untreated control cultures). The toxic effect of LPS was significantly counteracted by treatment with 10% *v*/*v* of RNS60 (94.23 ± 3.72%) (Fig. 1a–d). Cultures treated with ONS60, which has an oxygen concentration similar to RNS60 but has not been processed by TCP flow, did not show a difference compared to the NS conditions (Additional file 1: Figure S1). Therefore, we decided to omit ONS60 in the following experiments. Then, RNS60 was tested using an in vitro model of ALS consisting of astrocyte/spinal neuron co-cultures derived from C57BL/6J mouse embryos expressing human SOD1^G93A^ or NTG [35] (Fig. 1e–g). In this paradigm, a spontaneous and selective loss of large mutant motor neurons occurs after 6 DIV when compared with non-transgenic cultures under the same conditions [13, 35]. SOD1^G93A^ and NTG co-cultures were treated with 10% *v*/*v* of RNS60 or NS as control, at DIV0 and DIV3. At 6 DIV, SMI32-positive MNs were counted and the ratio of MNs to all NeuN-positive neurons was calculated. While MN viability in the untreated co-cultures expressing SOD1^G93A^ was only 55.38 ± 2.01% compared to the non-transgenic ones (NTG 100 ± 7.13%), it was significantly increased by treatment with RNS60 (92.76 ± 9.84%), while control solution NS did not modify MN survival (NS = 63.16 ± 4.70%) (Fig. 1i). Analysis of neuritic outgrowth of the MNs revealed that RNS60 significantly ameliorated the reduction of both the number and total extension of neurites in SOD1^G93A^ co-cultures, compared to NS (Fig. 1j, k).

### RNS60 delays neuromuscular impairment of C57BL/6-SOD1^G93A^ mice

**Fig. 2:**
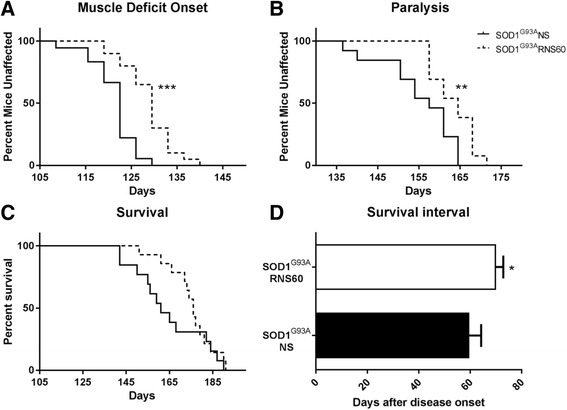
RNS60 delays muscle weakness onset and paralysis and increases survival interval in C57BL/6-SOD1^G93A^ mice. The treatment with RNS60 (300 μl/mouse/IP) every other day starting from the onset (hind limbs tremor and reduced abduction) **a** Kaplan-Meier curve showing the RNS60-induced delay (7 days) of the onset of muscle weakness based on the initial failure in the paw grip strength test for two consecutive time points. Log–rank Mantel–Cox test was done comparing NS (*n* = 21) vs RNS60 (*n* = 22) treated mice (*p* < 0.001). **b** Kaplan-Meier curve showing the RNS60-induced delay (7 days) of muscle paralysis, considered as the age at which the mice were no longer able to perform the grip strength test. Data were analyzed using the Log-rank test, *p* < 0.01. **c** Kaplan-Meier curve for survival showed an increase of about 10 days in mice treated with RNS60 (175.1 ± 2.8 days, mean ± SEM, *n* = 14 mice) compared to NS (164.7 ± 4.6 days, mean ± SEM, *n* = 13 mice) for the time at which 50% of the animals were euthanized. This increase was not statistically significant for the Log-rank test (*p* = 0.2544). **d** The survival interval, calculated from the symptom onset until the time at which the mice were euthanized, was increased by 17% by RNS60 (70.1 ± 2.9 days, mean ± SEM) compared to NS-treated mice (59.7 ± 4.6 days, mean ± SEM). The bar graph represents mean ± SEM. Difference between means was evaluated using non-parametric Mann Whitney test (*p* = 0.048)

Female C57BL/6-SOD1^G93A^ mice were randomly divided into two groups, one received NS and the other RNS60. All mice were treated intraperitoneally (IP) with RNS60 or NS (300 μL/mouse) every other day starting at 105 days, corresponding to about 1 week before the onset of muscle strength loss as we have routinely observed in these mice [42]. As shown in Fig. 2a, the RNS60 treatment significantly extended the age of the muscle strength impairment by 1 week (RNS60 128.8 ± 1.2 days, NS 121.3 ± 1.1 days, mean ± SEM, *p* < 0.0001). The age at which the mice were no longer able to perform the test was considered as the time of paralysis. Figure 2b shows that RNS60 treatment induced a significant delay of paralysis by about 8 days (163.4 ± 1.4 days, mean ± SEM) compared to NS (155.3 ± 2.5 days, mean ± SEM, *p* = 0.009). Consistently, the mean survival age was also extended by 10 days in RNS60-treated mice (175.1 ± 2.8 days, mean ± SEM) compared to the NS-treated group (164.7 ± 4.6 days, mean ± SEM), although this difference was not statistically significant (*p* = 0.254) (Fig. 2c). However, we observed that at the time when 50% (6/13) of mice treated with NS had died, more than 85% (12/14) of the mice treated with RNS60 were still alive. Overall, the mean duration from symptoms onset to end-stage sacrifice (survival interval) was increased in mice treated with RNS60 by about 17% (*p* = 0.048) (Fig. 2d).

### RNS60 prevents degeneration and impairment of spinal motor neurons and reduces the hSOD1 positive vacuoles

**Fig. 3:**
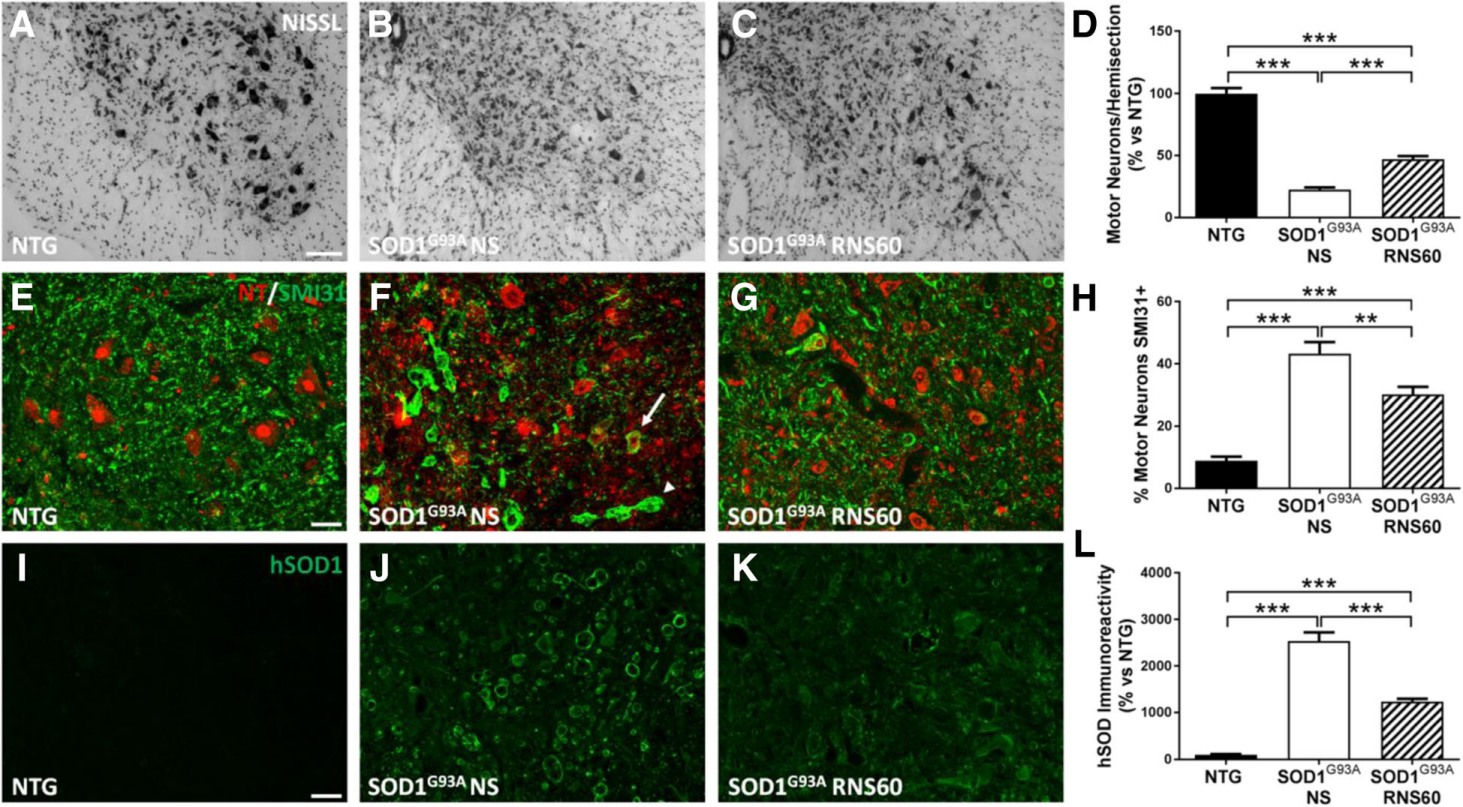
RNS60 prevents MN dysfunction, loss and reduces the hSOD1-positive vacuoles in C57BL/6-SOD1^G93A^ mice. **a**–**c** Representative images of NISSL staining performed on LSC sections from NTG and C57BL/6-SOD1^G93A^ mice treated with NS (SOD1^G93A^ NS) or RNS60 (SOD1^G93A^ RNS60) at the symptomatic stage of the disease (140 days). Scale bar: 100 μm. **d** Quantification of motor neurons with cell body area > 400 μm^2^ in LSC. At symptomatic stage of the disease, MNs were decreased in both transgenic groups of mice compared to NTG. Treatment with RNS60 displayed significant neuroprotection (46.9 ± 2.62%) compared to SOD1^G93A^ mice treated with NS (22.6% ± 1.7). The bar graph represents mean ± SEM as percentage versus NTG controls (*n* = 3 animals per group). **e**–**g** RNS60 reduced the accumulation of phosphorylated neurofilaments (SMI31 green) in the MN cell body (arrow) labeled with neurotrace (NT, red) and proximal axons (arrow head) which is an index of MN dysfunction (scale bar: 20 μm). **h** In transgenic mice, we observed a marked increase of SMI31-labeled MNs compared to NTG mice (8.9 ± 1.3%). RNS60 significantly reduced the number of impaired MN (30.1 ± 2.4%) compared to NS-treated SOD1^G93A^ mice (43.3 ± 3.7%). The bar graph represents mean ± SEM (*n* = 3 mice per group). **i**–**k** Laser scanning confocal photomicrographs of hSOD1 staining (green) in LSC MNs of NTG and transgenic mice treated with NS or RNS60 (scale bar: 20 μm). **l** Quantification showed the elevated level of hSOD1 immunoreactivity in transgenic LSC compared to NTG. This effect was significantly reduced by the treatment with RNS60. The bar graph represents mean ± SEM as a percentage versus NTG (*n* = 3 mice per group). All data were statistically analyzed using one-way ANOVA followed by post hoc Fisher’s LSD. **p < 0.01, ***p < 0.001

For the histopathological and biochemical analyses, a separate set of female C57BL/6-SOD1^G93A^ mice were randomly divided into two groups (NS or RNS60); these mice were sacrificed at 140 days of age corresponding to 80% muscle deficit of the untreated control group. A group of NTG littermates were also sacrificed at 20 weeks of age. The MNs remaining in the lumbar spinal cord of C57BL/6-SOD1^G93A^ mice were counted, and their viability was expressed as percentage of the MNs counted in NTG mice. While in C57BL/6-SOD1^G93A^ mice treated with NS only 22.6 ± 1.7% of MNs with a size > 400 μm^2^ were still present in the ventral horn of lumbar spinal cord, in the RNS60-treated mice, this number was significantly increased to more than twice (46.93 ± 2.62%) (Fig. 3a–d). Not only was the number of MNs higher, their function was protected too as demonstrated in Fig. 3e–h. Under normal conditions, phosphorylated neurofilament (NF) labeled with SMI31 antibody mostly stains the axons and neurites and is lacking from the MN soma (Fig. 3e). In contrast, when neurons become stressed or damaged, like in SOD1^G93A^ mice, phosphorylated NF strongly accumulates in the cell body as well as in swelled axons and neurites (Fig. 3f). In mice treated with RNS60, this stress-induced phenomenon appears clearly reduced compared with NS-treated mice (Fig. 3g). By counting the number of SMI31-positive substructures in MN somata and swelled neurites, we observed a significant reduction of these structures in RNS60-treated mice (30.1 ± 2.4%) compared to mice treated with NS (43.3 ± 3.7%) (Fig. 3h). Another common feature found in lumbar spinal cord of transgenic mice is the accumulation of hSOD1, which appears mainly confined to the membrane of vacuoles formed by the swelling of mitochondria [43, 44]. While in C57BL/6-SOD1^G93A^ mice treated with NS, we clearly observed numerous hSOD immunoreactive vacuoles in the lumbar spinal cord (Fig. 3j), their numbers were significantly reduced in RNS60 treated mice (Fig. 3k, l).

### RNS60 increases reactive gliosis and anti-inflammatory cytokines in the lumbar spinal cord

**Fig. 4:**
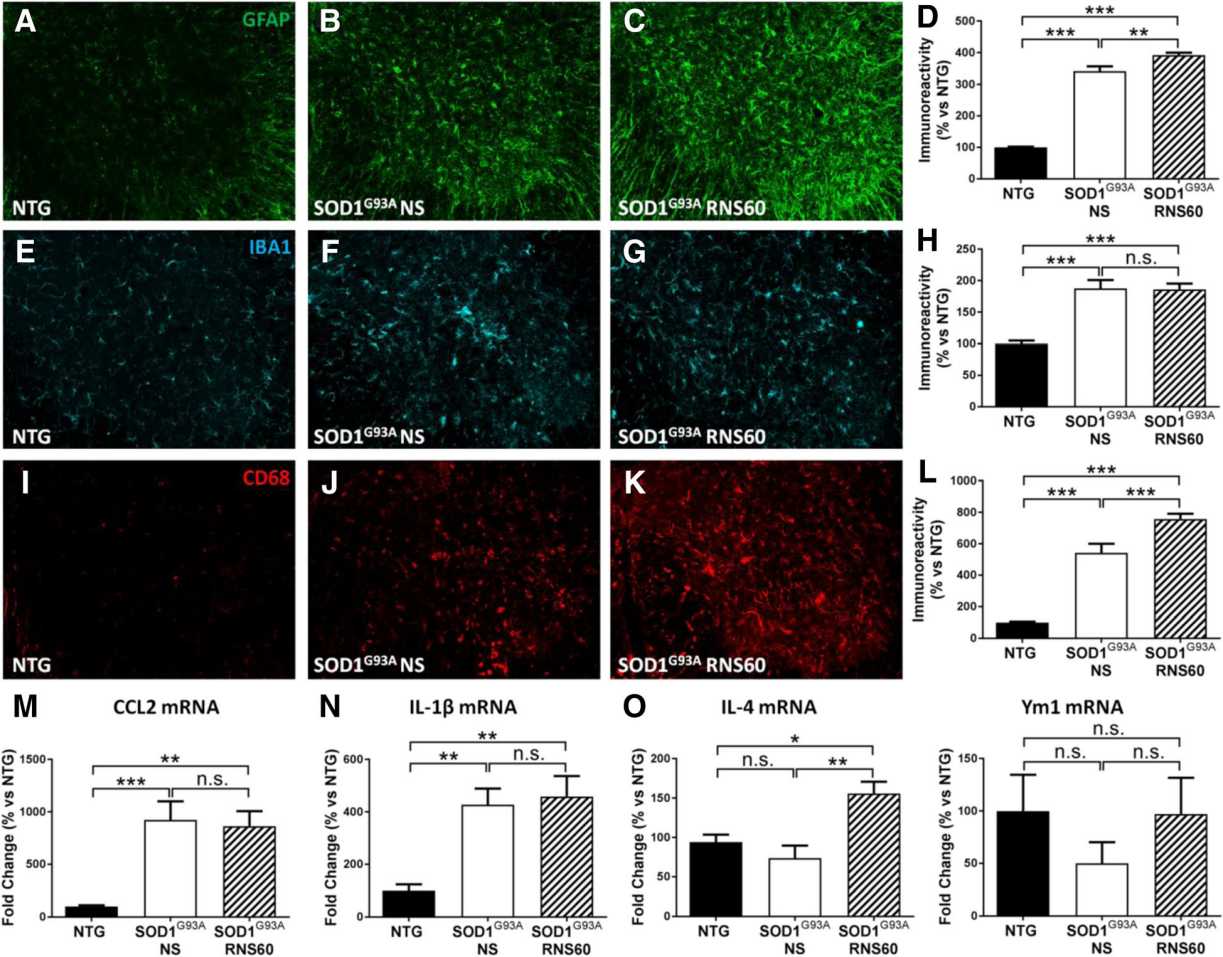
RNS60 activates astrocytes and phagocytic microglia and increases anti-inflammatory molecules in C57BL/6-SOD1^G93A^ mice. **a**–**c** Representative images of ventral LSC hemisections stained with GFAP (green) at 20 weeks. Scale bar: 50 μm. **d** Quantification of GFAP immunofluorescence showed elevated astrocytosis in the LSC ventral horn of C57BL/6-SOD1^G93A^ mice at the symptomatic stage of the pathology as compared to NTG expressed as 100%. Treatment with RNS60 significantly increased reactive astrocytosis. Bars are mean ± SEM as a percentage versus NTG controls (*n* = 3 animals for each group). **e**–**g** IBA1-stained (light blue) LSC hemisection from NTG, and SOD1^G93A^ mice treated with NS or RNS60. Scale bar: 50 μm. **h** Relative quantification showed higher level of IBA1 immunofluorescence in transgenic mice compared to NTG, without changes after the treatment with RNS60. Data are expressed as mean ± SEM as percentage versus NTG (*n* = 3 animals for each group). **i**–**k** CD68 positive macrophagic microglia (red) was labeled in ventral horn of LSC. Scale bar: 50 μm. **l** Immunoreactivity quantification showed an up-regulation of CD68 in the LSC of C57BL/6-SOD1G93A mice compared to NTG. This effect was exacerbated by the treatment with RNS60. The bar graph represents mean ± SEM as a percentage versus NTG controls (*n* = 3 mice per group). **m**–**p** Real-time PCR for pro-inflammatory (CCL2 and IL-1β) and anti-inflammatory (IL-4 and YM1) markers in the ventral portion of LSC of NTG mice expressed as 100% and transgenic mice treated with NS or RNS60. For CCL2 (*p* = 0.7516 SOD1^G93A^ RNS60 vs SOD1^G93A^ NS) and IL-1β (*p* = 0.7033 SOD1^G93A^ RNS60 vs SOD1^G93A^ NS) the increase observed in C57BL/6-SOD1^G93A^ mice treated with NS was unchanged by RNS60 treatment. On the contrary, as regard the anti-inflammatory markers such as IL-4 (*p* = 0.0033 SOD1^G93A^ RNS60 vs SOD1^G93A^ NS) and YM1 (*p* = 0.2861 SOD1^G93A^ RNS60 vs SOD1^G93A^ NS), we observed a clear increase in RNS60 treated mice suggesting an anti-inflammatory response, even if the increase of YM1 did not reach statistical significance. Data are normalized to β-actin and expressed as the mean ± SEM fold change ratio between the two transgenic mice groups as percentage versus NTG (*n* = 5 mice per group). All data were statistically analyzed using one-way ANOVA followed by post hoc Fisher’s LSD. *p < 0.05, **p < 0.01, ***p < 0.001, n.s. = non-significant

Reactive astrocytosis evaluated by GFAP immunoreactivity as well as microgliosis are considered hallmarks of neuroinflammation in the spinal cord of SOD1^G93A^ mice as they increase with the progression of the disease. In fact, we observed an increased immunoreactivity for the astroglial protein GFAP in the ventral horn of lumbar spinal cord of C57BL/6-SOD1^G93A^ mice treated with NS with respect to NTG mice. Unexpectedly, the treatment with RNS60 induced a slight but significant further increase of the GFAP immunoreactivity (Fig. 4a–d). This effect was less evident and not statistically significant when we evaluated the GFAP levels in the homogenate of the entire lumbar spinal cord by Western blot (Additional file 2: Figure S2). In addition, we did not detect changes in the levels of immunoreactivity for S100β, a marker of aberrant astrocytes that promotes MN degeneration in SOD1^G93A^ mice [45] (Additional file 3: Figure S3). With respect to microglia, we observed a differential response depending on the marker used to detect it. While reactive microglia immunostained with IBA1, which is high in C57BL/6-SOD1^G93A^ mice, was not changed by RNS60 treatment (Fig. 4e–h), macrophagic/phagocytic microglia labeled with CD68 antibody was increased in the ventral horn of lumbar spinal cord of RNS60 treated mice as compared to NS-treated mice (Fig. 4i–k). The relative quantification of the immune signal showed a significant upregulation in RNS60- versus NS-treated mice (Fig. 4l). When we measured transcript levels of the pro-inflammatory markers CCL2 (Fig. 4m) and IL-1β (Fig. 4n) in the ventral lumbar spinal cord of C57BL/6-SOD1^G93A^ mice, we found a significant increase compared to NTG mice that was not different between NS- and RNS60-treated mice. In contrast, the anti-inflammatory cytokine IL-4 (Fig. 4o) was significantly increased in RNS60-treated mice. Consistently, the reduction of the M2 marker YM1 observed in C57BL/6-SOD1^G93A^ mice compared to NTG was also prevented in RNS60-treated mice although the latter was not significant due to high variability of the data (Fig. 4p).

### RNS60 protects Schwann cells in the sciatic nerves of C57BL/6-SOD1^G93A^ mice

**Fig. 5:**
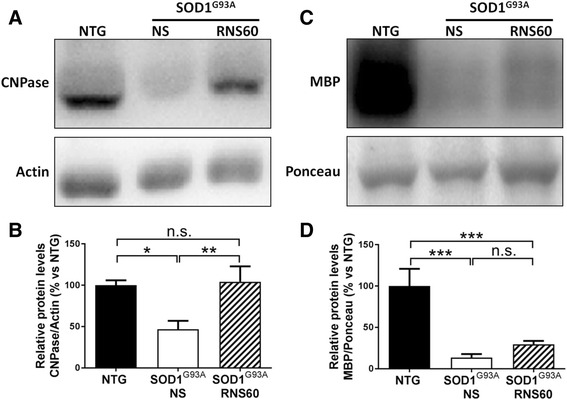
RNS60 preserves myelin maintenance in sciatic nerves of C57BL/6-SOD1^G93A^ mice. **a**–**d** Representative immunoblots of the typical myelin markers CNPase (**a**) and MBP (**c**) in sciatic nerve extracts from NTG and C57BL/6-SOD1^G93A^ mice treated with NS or RNS60 at symptomatic stage of the disease (140 days). **b**–**d** Densitometric analysis revealed a marked reduction of both CNPase and MBP in transgenic mice with respect to NTG. The treatment with RNS60 completely prevented the reduction of CNPase and slightly, but non-significantly (*p* = 0.332), counteracted the decrease of MBP. Data are reported as mean ± SEM of percentage vs NTG (*n* = 5 animals per group). Statistical analysis was performed using one-way ANOVA followed by post hoc Fisher’s LSD. *p < 0.05, **p < 0.01, ***p < 0.001, n.s. = non significant

Alterations of Schwann cells and consequent demyelination of peripheral nerves have been implicated in the disease progression in SOD1^G93A^ mice [19]. Since it has been demonstrated that RNS60 prevented loss of myelin in EAE mice and promoted survival, maturation, and expression of various myelin forming genes by oligodendrocytes in culture [30–32], we examined whether it may also promote myelin maintenance in the sciatic nerves of C57BL/6-SOD1^G93A^ mice. Figure 5a, b shows that the levels of CNPase, a myelin-associated enzyme, which is markedly reduced in the sciatic nerves of C57BL/6-SOD1^G93A^ mice treated with NS compared to NTG littermates, were completely restored by the treatment with RNS60. However, the reduction of myelin basic protein (MBP) levels in C57BL/6-SOD1^G93A^ mice was only modestly and not significantly attenuated by the treatment with RNS60 (Fig. 5c, d).

### RNS60 inhibits NMJ denervation and increases macrophage infiltration in tibialis anterior muscle

**Fig. 6:**
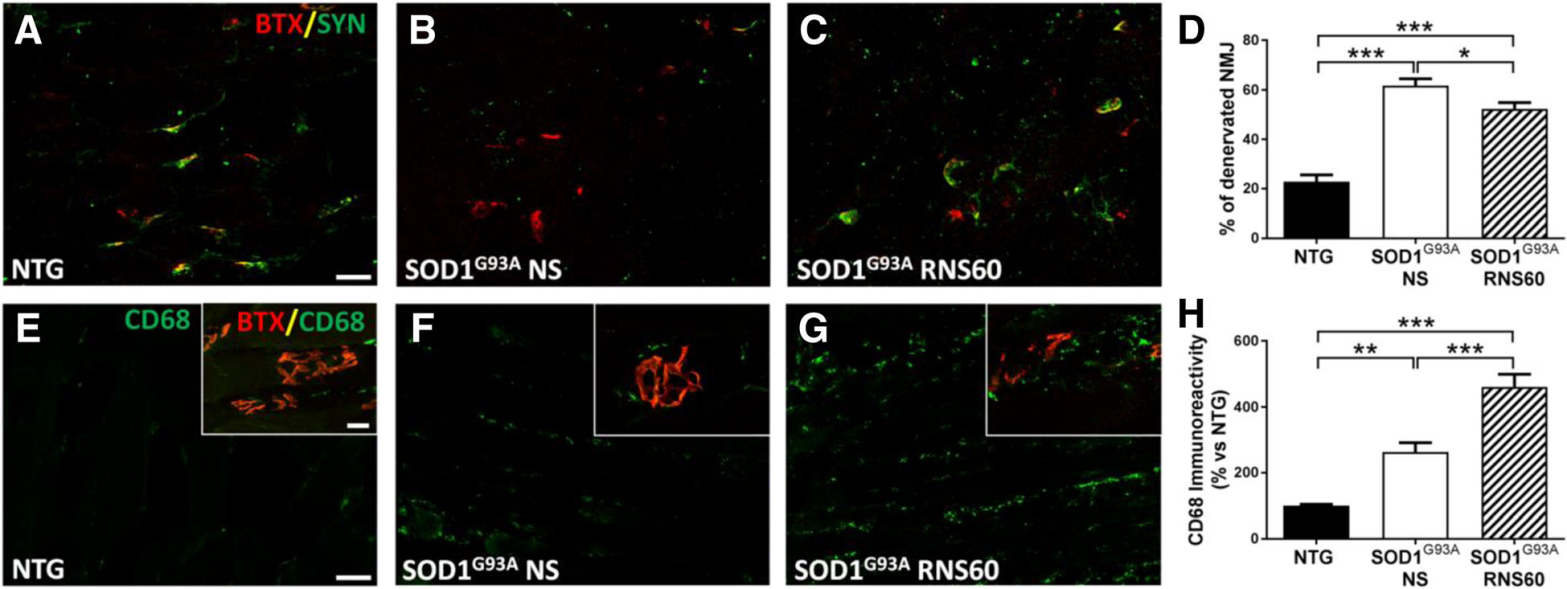
RNS60 reduces NMJ denervation and promotes macrophage recruitment in TAM of C57BL/6-SOD1^G93A^ mice. **a**–**c** Representative confocal images of co-localization of synaptophysin (SYN, green) with bungarotoxin (BTX, red) in TAM of NTG mice and C57BL/6-SOD1^G93A^ mice treated with NS or RNS60 (scale bar 50 μm). **d** Denervation of NMJ was higher in TAM of transgenic mice treated with NS as compared to NTG (61.8 ± 2.8% vs. 22.3 ± 2.75). Treatment with RNS60 slightly but significantly reduced this percentage (52.4 ± 2.5%). Data are expressed as mean ± SEM, (*n* = 5 animals per group). **e–g** Representative images of TAM of NTG, SOD1^G93A^ NS and SOD1^G93A^ RNS60 stained for CD68 (green), scale bar: 50 μm. Inserts show CD68+ cell infiltration at the level of NMJ labeled with bungarotoxin (red) at higher magnification, scale bar: 10 μm. **h** The quantification of immunoreactivity showed an increase of CD68+ cells in transgenic mice treated with NS compared to NTG. The treatment with RNS60 exacerbated the recruitment of macrophages. Data are expressed as mean ± SEM, (*n* = 5 animals per group). All data were statistically analysized using one-way ANOVA followed by post hoc Fisher’s LSD, *p < 0.05, **p < 0.01, ***p < 0.001

We recently demonstrated that neuromuscular denervation, more than motor neuron loss, contributes to an aggressive disease phenotype in SOD1^G93A^ mice [19, 20, 46]. Therefore, we examined the effect of RNS60 on the level of NMJ denervation in tibialis anterior muscle (TAM) sections from 20-week-old C57BL/6-SOD1^G93A^ mice using immunoreactivity for synaptophysin and bungarotoxin as pre-synaptic and post-synaptic terminal markers respectively (Fig. 6a–c). The numbers of denervated plaques in mice treated with either NS or RNS60 were calculated as percentage of those found in NTG littermates. As shown in Fig. 6d, the number of denervated plaques in mice treated with RNS60 was slightly but significantly lower compared to those of NS-treated mice. We also investigated the level of macrophage infiltration in the muscles which plays a role in modulating the muscle reinnervation [47]. We observed an increased immune staining for CD68 in C57BL/6-SOD1^G93A^ mice treated with NS compared with NTG littermates and this effect was exacerbated by the treatment with RNS60 (Fig. 6e–h).

### RNS60 reduces oxidative damage and increases anti-oxidant response in the lumbar spinal cord

**Fig. 7:**
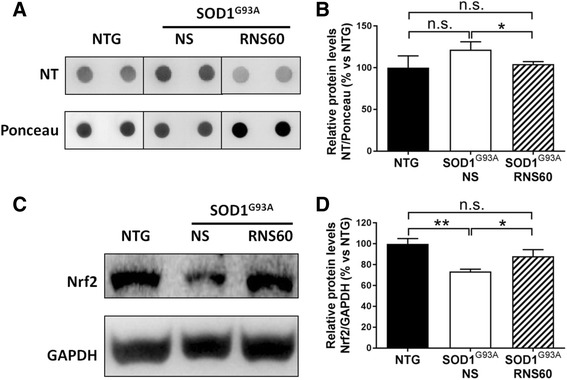
RNS60 increases anti-oxidant response and reduces oxidative stress in the lumbar spinal cord of C57BL/6-SOD1^G93A^ mice. Representative dot-blot (NT) or immunoblots (Nrf2) and relative quantification performed on the ventral portion of LSC of NTG mice or transgenic C57BL/6-SOD1^G93A^ mice treated with NS or RNS60, at 20 weeks of age. **a**, **b** The levels of NT are increased in C57BL/6-SOD1^G93A^ mice treated with NS compared to NTG mice, whereas they are similar to NTG in mice treated with RNS60. **c**, **d** While the levels of Nrf2 are reduced in C57BL/6-SOD1^G93A^ mice treated with NS compared to NTG mice, the treatment with RNS60 prevented this reduction. Data are expressed as mean ± SEM, (n = 5 animals per group). All data were statistically analyzed using one-way ANOVA followed by post hoc Fisher’s LSD. *p < 0.05, **p < 0.01, ***p < 0.001, n.s. = non significant

Oxidative stress associated with mitochondrial dysfunction is a primary cause of neurodegeneration in ALS. In particular nitrosative stress, due to peroxynitrite-mediated tyrosine nitration contributes to the irreversible protein damage [48]. Nitrotyrosine (NT) is a marker of this aberrant process in ALS patients as well as in ALS mouse models [41]. Since putative mechanisms of RNS60’s action include induction of mitochondrial biogenesis [49] and the inhibition of NO production from activated microglia [22], we examined its effect on the levels of NT in the ventral lumbar spinal cord of C57BL/6-SOD1^G93A^ mice. We found that the levels of NT were significantly reduced in mice treated with RNS60 compared to those treated with NS (Fig. 7a, b). Interestingly, when we examined the levels of nuclear erythroid 2-related factor 2 (Nrf2), a key mediator of the anti-oxidant response Nrf2 was observed to be reduced in the spinal cord of NS treated C57BL/6-SOD1^G93A^ mice compared to NTG mice and the loss was significantly counteracted by treatment with RNS60 (Fig. 7c, d).

### RNS60 stimulates the activation of the PI3K-Akt pro-survival pathway in spinal cord and sciatic nerves

**Fig. 8:**
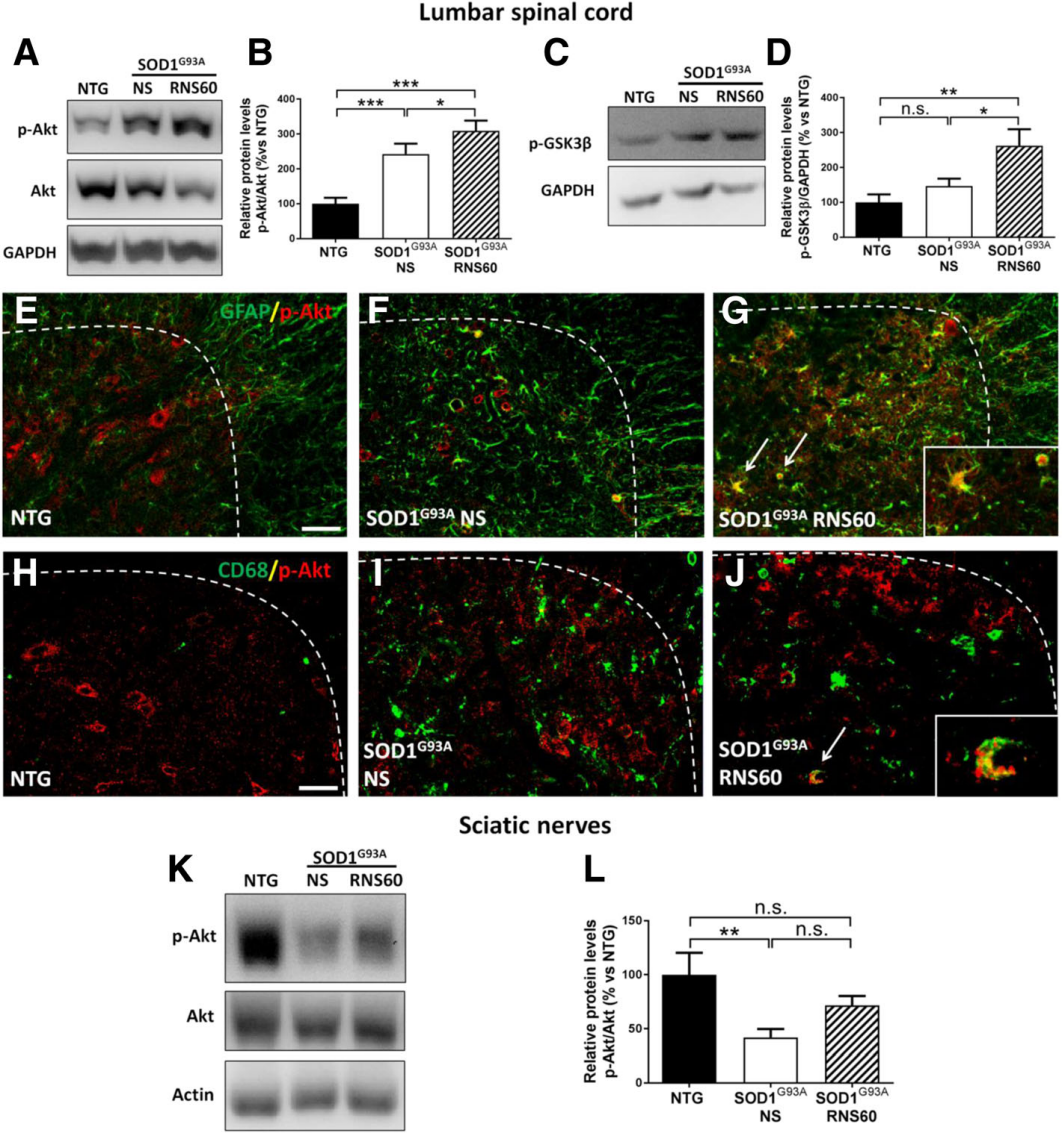
RNS60 activates the p-Akt pro-survival pathway in the LSC and sciatic nerves of C57BL/6-SOD1^G93A^ mice. **a**, **b** Representative immunoblots performed on the ventral portion of LSC of NTG mice or transgenic C57BL/6-SOD1^G93A^ mice treated with NS or RNS60, at 20 weeks of age, and relative quantification. There was an increase of Akt phosphorylation (at Ser473) in RNS60 treated mice compared to NS treated mice. This was paralleled by increased phosphorylation of GSK3β at Ser9 (**c**, **d**), one of the downstream targets of activated Akt. **e**–**g** Representative images of the co-localization of p-Akt and GFAP immunostaining. P-Akt (red) was highly expressed specifically in the MNs of NTG mice (**e**). In SOD1^G93A^ mice treated with NS, the number of p-Akt labeled MNs decreased while few of the highly reactive astrocytes (green) co-localized with p-Akt (**f**). Colocalization of Akt and GFAP was exacerbated in the SOD1^G93A^ mice treated with RNS60 (G, arrows). The inset in G show enlarged reactive astrocytes expressing p-Akt and showing a spheroid or less ramified morphology (*n* = 3 animals per group). **h**–**j** Representative images of the co-localization of p-Akt (red) and CD68 (green) immunostaining. In SOD1^G93A^ mice treated with RNS60, few CD68-positive microglial cells colocalized with p-Akt (arrow), while this phenomenon was not detected in SOD1^G93A^ mice treated with vehicle (**i**). The inset in J show enlarged macrophagic-microglial cells expressing p-Akt (*n* = 3 animals per group). **k**, **l** Decreased levels of Ser473 Akt phosphorylation were found in the sciatic nerves of SOD1^G93A^ mice treated with NS but not in RNS60 treated mice compared to NTG. Bar graphs represents mean ± SEM as a percentage versus NTG controls (n = 5 animals per group). All data were statistically analyzed using one-way ANOVA followed by post hoc Fisher’s LSD. *p < 0.05, **p < 0.01, ***p < 0.001, n.s. = not significant

To investigate the possible mechanism through which RNS60 protects MNs and NMJs in C57BL/6-SOD1^G93A^ mice, we examined the activation of the Akt pro-survival pathway by Western blot analysis of the ventral horn of lumbar spinal cord and sciatic nerves using an antibody specific for Akt phosphorylated at Ser473. Levels of activated, phosphorylated Akt (p-Akt) were found to be significantly increased in the spinal cord of C57BL/6-SOD1^G93A^ mice compared to NTG mice, and this effect was further increased by the treatment with RNS60 (Fig. 8a, b). Similarly, the levels of glycogen synthase kinase 3beta (GSK3β) phosphorylated at Ser 9, a substrate of activated Akt [50], were also found to be significantly increased in C57BL/6-SOD1^G93A^ mice treated with RNS60 compared to those treated with NS (Fig. 8c, d). Interestingly, analyzing the p-Akt distribution by immunohistochemistry in the ventral of horn spinal cord, we observed a partial co-localization of p-Akt with GFAP reactive astrocytes in SOD1^G93A^ mice compared to NTG mice (Fig. 8e, f) and this phenomenon was exacerbated in the reactive astrocytes of RNS60 treated (Fig. 8g, arrows) compared to NS-treated SOD1^G93A^ mice. The percent of astrocytes labeled with p-Akt over the total number of GFAP-positive cells in the hemisection of ventral horn of lumbar spinal cord were 9.8 ± 1.3 and 17.6 ± 2.0 (mean ± SEM; *p* < 0.05) in NS- and RNS60-treated mice, respectively. In addition, most of these labeled astrocytes showed a round-shaped morphology with little or no ramification. In C57BL/6-SOD1^G93A^ mice treated with RNS60, we also found some CD68-positive microglial cells co-localized with p-Akt (Fig. 8j, arrow), while this phenomenon was not detected in SOD1^G93A^ mice treated with NS (Fig. 8i). Some surviving MNs were also markedly labeled for p-Akt (data not shown).

Unlike in the spinal cord, we observed that the levels of p-Akt were significantly reduced in sciatic nerves of C57BL/6-SOD1^G93A^ mice compared to NTG, and this effect was also partially rescued by the treatment with RNS60 (Fig. 8k, l).

### RNS60 enhances the Treg population in the spleen of SOD1^G93A^ mice but not in the lumbar spinal cord

**Fig. 9:**
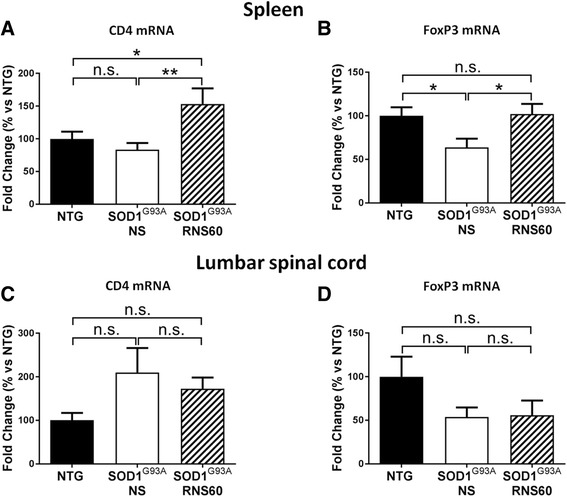
RNS60 augments Treg population in the spleen but not in LSC of C57BL/6-SOD1^G93A^ mice. **a**, **b** Real-time PCR for typical markers of Treg in spleen of SOD1^G93A^ mice expressed as 100% and transgenic mice treated with NS or RNS60. Treatment with RNS60 completely reverted the decrease of FoxP3 found in the spleen of SOD1^G93A^ NS mice compared to NTG mice and markedly increased the levels of CD4+ T cells with respect to both the SOD1^G93A^ NS and NTG mice. **c**, **d** On the contrary, we did not observe a significant difference for both markers between the two groups of transgenic mice in the LSC. Data are normalized to β-actin and expressed as the mean ± SEM change ratio between the two group of transgenic mice as percentage versus NTG (*n* = 5 mice per group). All data were statistically analyzed using one-way ANOVA followed by post hoc Fisher’s LSD. *p < 0.05, **p < 0.01, ***p < 0.001, n.s. = non-significant

Emerging evidence suggests that low levels of regulatory T lymphocytes (Tregs) are responsible for ALS disease progression in patients and mice [12, 18, 51]. Since RNS60 was reported to inhibit the disease progression in EAE mice by increasing the Treg population [30, 31], we examined the proportions of Tregs both in the spinal cord and in the spleen, the peripheral source of these cells, in SOD1^G93A^ mice. Treg levels were assessed by measuring transcript expression levels of specific markers of these cells, FoxP3 and CD4, using real-time PCR. The treatment with RNS60 significantly increased the levels of CD4 and prevented the significant decrease of FoxP3 mRNA found in the spleen of SOD1^G93A^ mice treated with NS with respect to NTG littermates (Fig. 9a, b). In the spinal cord, however, we observed only non-significant changes of CD4 and FoxP3 mRNA in the C57BL/6-SOD1^G93A^ mice compared to NTG littermates, and the levels were not affected by RNS60 treatment (Fig. 9c, d,).

### RNS60 neither ameliorates disease progression nor protects MNs in 129Sv-SOD1^G93A^ mice

**Fig. 10:**
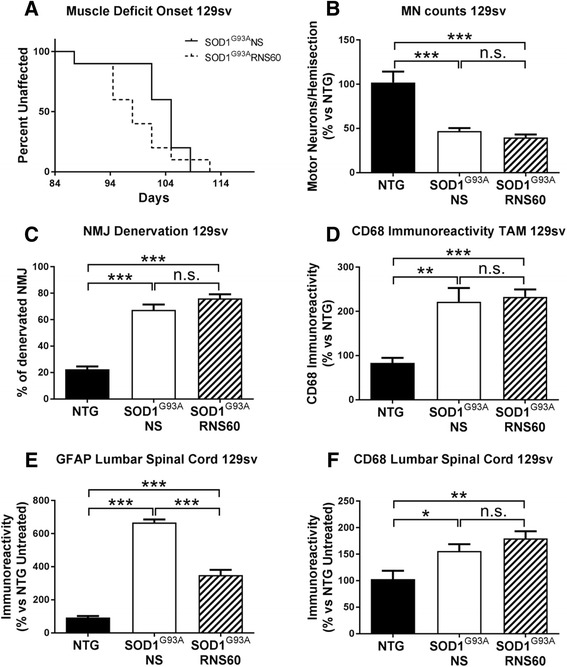
RNS60 does not affect the disease progression and the pathology in 129Sv-SOD1^G93A^ mice. Treatment with RNS60 or NS (300 μl/mouse/IP) every other day started from the onset (91 days, hind limbs tremor and reduced abduction) until the symptomatic phase (112 days). **a** Kaplan-Meier curve shows that the treatment with RNS60 does not modify the onset of muscle strength impairment in fast progressive mice. The curve was evaluated by the Log-rank test, *p* = 0.1886 (*n* = 10 NS; n = 10 RNS60). **b** Quantification of motor neurons with cell body area > 400 μm^2^ in LSC. At symptomatic stage of the disease, MNs decreased in both SOD1^G93A^ groups of mice compared to NTG without any difference between the treatments. The bar graphs represent mean ± SEM as percentage versus NTG controls (*n* = 5 animals per group). **c** Quantification of NMJ denervation in TAM. In transgenic mice there is a higher percentage of denervated plaques compared to NTG. The treatment with RNS60 does not modify these numbers. Data are expressed as mean ± SEM, (*n* = 5 animals per group). **d** Quantification of immunoreactivity showed an increase of CD68+ cells in transgenic mice compared to NTG without any change by RNS60 treatment. Data are expressed as mean ± SEM, (*n* = 5 animals per group). **e** Quantification of immunofluorescence showed elevated astrocytosis in the LSC ventral horn of 129Sv-SOD1^G93A^ mice at the symptomatic stage of the pathology as compared to NTG expressed as 100%. Treatment with RNS60 significantly decreases the reactive astrocytosis (*p* < 0.001 SOD1^G93A^ RNS60 vs SOD1^G93A^ NS). **f** CD68 immunoreactivity quantification showed an up-regulation in the LSC of SOD1^G93A^ mice compared to NTG. This effect was unchanged by the treatment with RNS60. Bar graphs are mean ± SEM as percentage versus NTG (*n* = 5 animals for each group). All data were statistically analyzed using one-way ANOVA followed by post hoc Fisher’s LSD. *p < 0.05, **p < 0.01, ***p < 0.001, n.s. = non-significant

Recently, we characterized a variant of the SOD1^G93A^ mouse model (129Sv-SOD1^G93A^) expressing the same amount of mutant hSOD1 but showing an earlier onset and a more rapid disease progression compared to C57BL/6-SOD1^G93A^ mice [20, 21, 46]. Among the features that differentiate the two mouse models, we found a massive downregulation of mitochondria-related transcripts, decreased ATP levels and a lower activation of immune response-related molecules in the motor neurons of 129Sv-SOD1^G93A^ mice [21]. This was associated with an earlier and stronger NMJ denervation and with a poor activation of immune cells in the sciatic nerves and muscles compared to C57BL/6-SOD1^G93A^ mice [19]. To further test the therapeutic mechanism of RNS60, we investigated its effect on the disease onset of 129Sv-SOD1^G93A^ mice (Fig. 10a) as well as on the MNs count (Fig. 10b), NMJs denervation (Fig. 10c), and activation of peripheral macrophage response (Fig. 10d). Interestingly, no differences were observed between RNS60 and NS treatment in 129Sv-SOD1^G93A^ mice in any of the parameters analyzed. On the contrary, in the spinal cord, unlike in C57BL/6-SOD1^G93A^ mice, we found reduced levels of GFAP and no difference in the upregulation of CD68 in response to RNS60 treatment compared to NS (Fig. 10e, f).

## Discussion

The current work demonstrates, for the first time, therapeutic effects of RNS60 in cellular and animal models of ALS. RNS60 is a novel experimental drug that contains charge-stabilized nanostructures consisting of an oxygen nanobubble core surrounded by an electrical double-layer at the liquid/gas interface. The biological effects of RNS60 have been attributed to the physical properties of the charge-stabilized nanostructures as ONS60, a control solution containing levels of oxygen comparable with RNS60 (50 to 60 ppm) but not subjected to TCP flow had not been effective in several earlier studies [22, 24–26]. We confirmed this observation in the model of motor neuron/glia co-cultures exposed to LPS (Additional file 1: Figure S1).

**Fig. 11:**
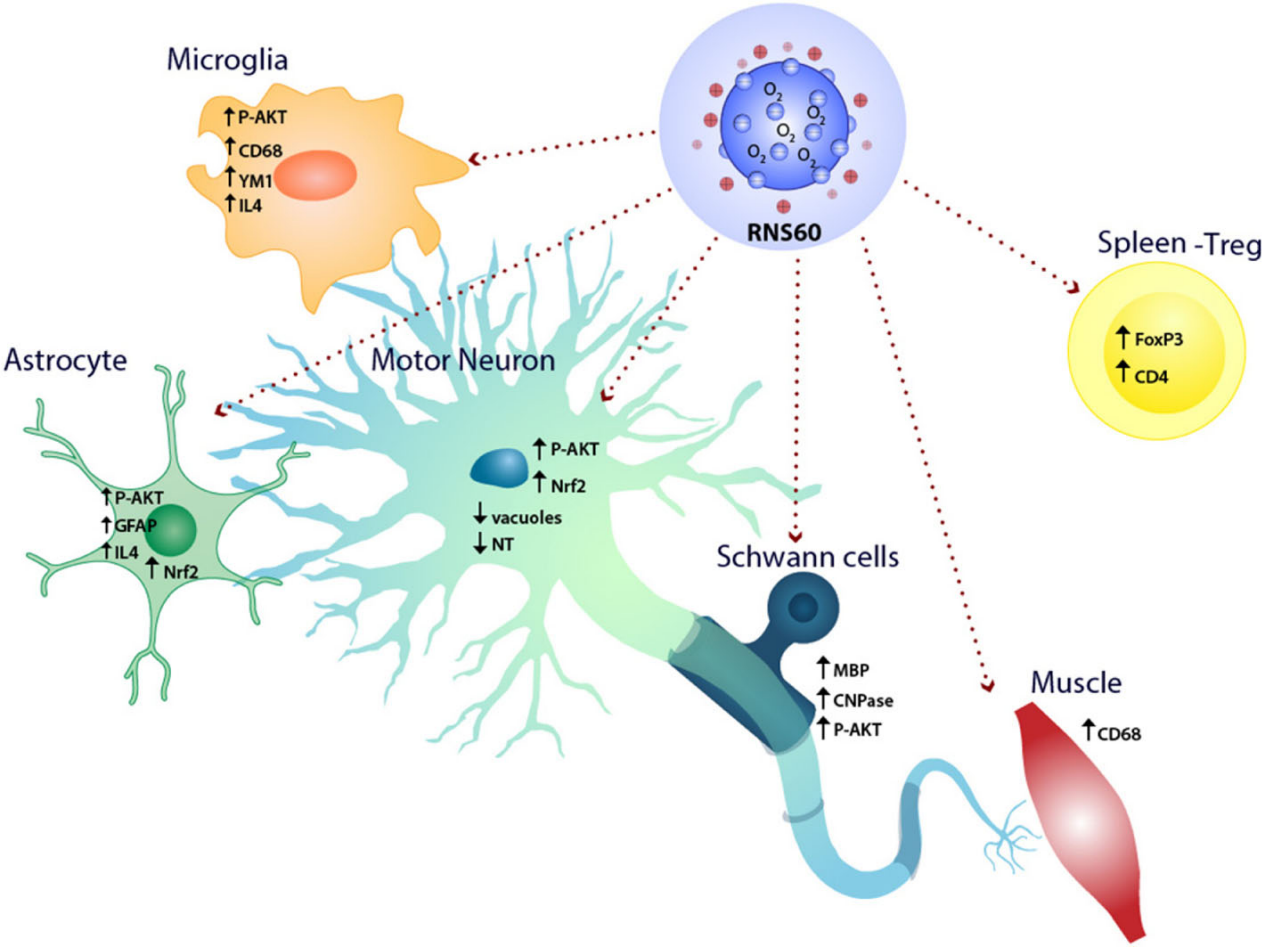
Schematic view of the potential mechanisms by which RNS60 protect motor neurons and ameliorate the neuromuscular impairment in ALS. In the motor neurons RNS60 activates the p-Akt mediated pro-survival pathway and prevents the decrease of Nrf2 mediated anti-oxidant response while reducing the mitochondrial alterations (reduced vacuoles) and oxidative stress (NT). RNS60 also enhances phosphorylation of p-Akt, and levels of Nrf2, in a subpopulation of protective astrocytes that lead to an overexpression of the IL-4. IL-4 then induces the polarization of CD68 microglia toward a prohealing M2 phenotype creating a neuroprotective environment. In the sciatic nerve RNS60 prevents demyelination by protecting Schwann cells through the p-Akt pro-survival pathway and this results in the reduced NMJ denervation. This, together with the increased of CD68 positive macrophage recruitment in the muscle and the consequent debris clearing and tissue remodeling, results in the amelioration of neuromuscular impairment. RNS60 may also exert its protective action through the activation of immunomodulatory Treg, known to be negatively correlated with the severity of ALS in patients and animal models

RNS60’s putative mechanism of action encompasses the activation of pro-survival pathways, increase in mitochondrial biogenesis, myelin maintenance and repair, reduction of inflammation, and increase of Tregs [22–32]. The current work demonstrates that RNS60 has a beneficial effect in cellular and animal models of ALS presumably through the same mechanisms of action (Fig. 11).

Initially, we found that RNS60 partially protects the motor neuron damage induced by LPS-activated microglia, suggesting an anti-inflammatory property of this potential therapeutic. This is consistent with previous studies showing that RNS60 suppressed the LPS-induced expression of iNOS in microglia [22]. Next, we demonstrated that RNS60 protected motor neurons (MN) in astrocytes-spinal neurons co-cultures carrying the SOD1^G93A^ mutation.

In addition, and most importantly, intraperitoneal treatment of C57BL/6-SOD1^G93A^ mice with RNS60 beginning at the onset of disease symptoms resulted in significantly improved outcome, which was characterized by protection of motor neurons, a delay in the onset of neuromuscular deficit and paralysis, and an increase of the mean survival interval from onset. Neuroprotective effects of RNS60 treatment were seen previously in other models of neuroinflammatory diseases, in particular the MPTP mouse model of PD [23]. The protective effects of RNS60 in this model, as well as in the case of microglial iNOS expression and NF-kB activation by LPS in vitro [22], were dependent on the activation of the class 1A PI3K-Akt pathway, which has emerged as a central mechanism of action of RNS60.

The effects seen in our study are likely mediated through the same pathway based on the following observations. Using the experimental paradigm of the in vitro experiments presented here, we have previously demonstrated that MN death was counteracted by Akt activation-induced motor neuron-specific overexpression of MyrAKT3 [13]. Involvement of Akt is further supported by the fact that RNS60 also preserved the number and length of neurites, which is consistent with a previous study reporting RNS60 to increase the number of neurites as well as the length, size, and maturation of dendritic spines in primary hippocampal neurons through the activation of the PI3K-Akt pathway [26]. The most direct evidence comes from the C57BL/6-SOD1^G93A^ mouse model, where we measured increased phosphorylation of Akt in lumbar spinal cord and sciatic nerves of mice treated with RNS60. Increased Akt phosphorylation was paralleled by phosphorylation (inactivation) of GSK3-β, further confirming the functional activation of Akt. The activation of this pathway also has previously been linked to motor neuronal protection in vivo. For example, we reported that the specific induction of Akt3 via lentiviral vector in motor neuron sustained cell viability via inhibition of GSK3β (phosphorylated at Ser9) [13]. Moreover, delivery of insulin-like growth factor 1 (IGF-1) to SOD1^G93A^ mice through motor neuron-targeted viral vectors [15], or by intrathecal infusion [52], partially protected motor neurons and delayed disease progression via activation Akt in the spinal cord. Similarly, intracerebroventricular injection of VEGF in SOD1^G93A^ rats also improved motor performance, prolonged survival, and raised p-Akt levels in the spinal cord [17, 53].

Unexpectedly, in RNS60-treated C57BL/6-SOD1^G93A^ mice, higher level of p-Akt was present not only in viable motor neurons but also by GFAP-positive reactive astrocytes in the ventral horn of lumbar spinal cord at the time when the motor neurons were protected, and muscle paralysis was delayed. This may appear counterintuitive because reactive astrocytosis is considered a hallmark of neuronal death. It has been demonstrated, however, that not all astrocytes are intrinsically neurotoxic for motor neurons as the total ablation of proliferating astrocytes did not slow the disease progression and motor neuron loss in SOD1 mutant mice [54]. Likewise, a focal degeneration of astrocytes has been reported in concomitance with the loss of motor neurons in SOD1^G93A^ mice, and their protection through metabotropic GluR5 receptor inhibition significantly delayed the onset of the disease [55]. These protective astrocytes appeared as spheroid GFAP-positive cells that existed together with typical reactive astrocytes [55]. Interestingly, many p-Akt-positive astrocytes in RNS60-treated mice appeared with similar spheroid morphology. This suggests that RNS60 may have prevented the degeneration of these focal astrocytes through the activation of p-Akt, thus promoting motor neuron survival. Since the level of the astrocyte specific glutamate transporter 1 (GLT1), a key factor in the control of excitotoxicity, is increased by the activation of the Akt pathway [56], this could be a potential mechanism for the MN protective effect of RNS60. The exact mechanism by which RNS60 activates Akt in astrocytes is presently unknown. Studies in vitro have demonstrated that IGF1 is a potent activator of Akt in astrocytes during oxidative stress and that this is a key mechanism for IGF1 to induce neuroprotection against oxidative stress injury [57]. Thus, it is possible that even in the presence of the pro-oxidant SOD1^G93A^, the activated astrocytes in the spinal cord of RNS60-treated mice may acquire a neuroprotective function through the activation of Akt. Whether this may be mediated by IGF1 or other factors needs to be investigated.

In addition to the increase of astrocytes, the number of CD68-positive microglia in the spinal cord of RNS60-treated C57BL/6-SOD1^G93A^ mice was also increased. CD68 is expressed on phagocytic cells that can adopt a protective M2 phenotype in response to injection of anti-inflammatory IL-4 [58]. Interestingly, we detected a concomitant increase of IL-4 in the lumbar spinal cord of RNS60-treated mice. In addition, the reduction of Ym1, a marker of the M2 microglia phenotype in SOD1^G93A^ mice, was reverted by the treatment with RNS60 while the pro-inflammatory cytokines IL-1β and CCL2 remained unchanged. We also observed activation of Akt in CD68-positive microglial cells only in RNS60-treated mice. This is consistent with the observation that the PI3K/Akt pathway and its downstream targets have emerged as central regulators of M2 phenotype activation in macrophages/microglia [59]. For example, HDAC inhibitors have been shown to polarize microglia toward M2 by enhancing PI3K/Akt signaling in a model of severe traumatic brain injury preventing white matter damage [60]. Interestingly, Akt activation in macrophages was associated with reduced M1 markers and disease severity in a mouse model of EAE [61].

The neuroprotective role of M2 microglia is still debated but it seems that an increase of macrophagic activity in a specific phase of the disease may be associated with debris clearing and tissue remodeling [62, 63]. This process is particularly relevant at the level of the peripheral nervous system where immune cell infiltration may promote the phagocytic activity of Schwann cells and macrophages toward the degenerated axons to permit functional nerve regeneration and NMJ innervations [64–66]. We have recently demonstrated that the recruitment of immune cells in the peripheral nervous system delays muscle denervation and prolongs the lifespan in C57BL/6-SOD1^G93A^ mice [19]. Here, we found that RNS60 increased macrophage recruitment in tibialis anterior muscle and this correlated with reduced NMJ denervation.

Consistent with a protective role of immune cells, 129Sv-SOD1^G93A^ mice showing a fast disease progression are less prone to recruit macrophages and T cells in the peripheral sciatic nerve at the disease onset [20]. We therefore hypothesize that RNS60’s ineffectiveness in delaying onset and progression of muscular deficit and motor neuron degeneration in 129Sv-SOD1^G93A^ mice was due to the lack of response to RNS60-induced activation of the protective immune response. This is consistent with the lack of increase of CD68 immunoreactivity in the muscle tibialis and spinal cord in these mice compared to RNS60-treated C57BL/6 strain, and with a reduction of GFAP immunoreactivity in the spinal cord compared to NS-treated mice. This hypothesis is also in agreement with the observation of a lower macrophage recruitment in 129Sv mice, compared to C57BL/6 mice, after acute spinal cord injury [67]. In addition, the lower immune response in 129Sv mice is also reflected by the fact that these mice, in contrast to C57BL/6 mice, are resistant to EAE induced by myelin oligodendrocyte glycoprotein (MOG) [68]. We recently demonstrated that the comparison of 129Sv-SOD1^G93A^ and C57BL/6-SOD1^G93A^ mouse strains is useful to study disease susceptibility and to test novel therapeutic approaches aimed at addressing disease heterogeneity [69]. This may be relevant for the use of RNS60 in clinical trials of ALS patient cohorts that show heterogeneous disease severity. For example, a recent clinical trial with edaravone showed efficacy only in a well-defined group of patients [5]. A deeper biochemical and molecular analysis of these two mouse models might help to identify potential biomarkers to predict the positive or negative response to treatment in clinical trials.

In further support of potential protective immunomodulatory mechanisms, RNS60 increased Tregs in the spleen of C57BL/6-SOD1^G93A^ mice. Tregs are increased at early slow progressing stages of ALS and decreased during the rapidly progressing phase both in mice and in rapidly progressing ALS patients [18]. Passive transfer of endogenous Treg from ALS mice in the early disease stage into recipient ALS mice in a more progressed stage, without ex vivo activation, were shown to sustain IL-4 levels and M2 microglia, lengthen disease duration, and prolong survival [14, 18, 70]. We observed increased levels of IL-4 and a tendency to increase of the M2 microglia marker YM1 in the spinal cord of C57BL/6-SOD1^G93A^ mice treated with RNS60, despite the fact that the expression levels of CD4 T cells and FoxP3 were unchanged in this compartment. Thus, we hypothesize that peripheral Treg may contribute to the protective effect of RNS60 through a possible action at the neuromuscular level; however, the mechanism involved needs to be investigated.

Schwann cells are linked to the pathology in C57BL/6-SOD1^G93A^ mice and seem to contribute to the protective action of RNS60. We found a significant downregulation of p-Akt in the sciatic nerves of symptomatic C57BL/6-SOD1^G93A^ mice, which was associated with a marked decrease of both CNPase and MBP. This supports the prior finding of a key role of Akt in regulating the PNS myelination, enhancing membrane wrapping and myelin protein synthesis [71–75]. The downregulation of p-Akt, CNPase, and MBP was inhibited or reverted by treatment with RNS60. This is in line with the evidence that RNS60 retained the expression of myelin genes and blocked demyelination in CNS of EAE mice through the promotion of oligodendrocyte survival and OPC differentiation [30–32]. A more recent report showed a direct transcriptional effect of RNS60 on expression of myelin-related genes via the activation of PI3K-Akt-CREB [24].

RNS60 has been reported to modulate mitochondrial bioenergetics. In oligodendrocytes subjected to metabolic stress, RNS60 enhances the capacity of mitochondria to produce ATP [32]. In a neuronal cell line, primary dopaminergic neurons, and in the nigra of MPTP challenged mice, RNS60 was reported to upregulate mitochondrial biogenesis [49]. Alterations in the structure and function of mitochondria are a typical trait of ALS. In particular, the expression of mutant SOD1 is causally linked to alteration in their motility, dynamics, and turnover leading to energy deficit, calcium mishandling, and oxidative stress, which are all key contributors to motor neuron death [76]. In fact, it is known that mutant SOD1 accumulates in the outer membrane of mitochondria and causes the swelling of these membranes and consequent vacuole formation [43, 44, 77]. In support of an involvement of mitochondria in the effects of RNS60 observed in this study, we found a reduction of vacuoles immunostained for mutant hSOD1 in the spinal cord of RNS60-treated C57BL/6-SOD1^G93A^ mice compared to the NS-treated mice. That the protective effect of RNS60 could be partly mediated by action on mitochondria is also indicated by the fact that RNS60 is ineffective in 129Sv-SOD1^G93A^ mice. In fact, the pathological phenotype of these mice, unlike that of the C57BL/6-SOD1^G93A^ mice, is largely due to a marked mitochondrial dysfunction that could render them unresponsive to the protective action of RNS60.

How the mitochondrial effects of RNS60 are related to the modulation of the PI3K-Akt pathway is unclear at this time. Reports on RNS60-induced increased synaptic transmission by upregulated mitochondrial ATP synthesis in a squid giant synapse ex vivo model [27, 28] have raised the notion that delivery of the oxygen contained in the nanobubbles may directly feed into mitochondrial ATP production [27]. Of note though, RNS60 has been reported to increase mitochondrial biogenesis and ATP generation in a neuronal cell line as well as in primary dopaminergic neurons in a PI3K-dependent manner, lending support to an Akt-mediated process [49]. In other cell types, Akt has been shown to be involved in the regulation of bioenergetics. In cultured hepatocytes, for example, activated Akt translocates to the mitochondria where it phosphorylates subunits of ATP synthase and GSK3-β [78]. Akt-induced inhibition of GSK3-β in turn leads to activation of the mitochondrial enzyme pyruvate dehydrogenase (PDH), thereby boosting mitochondrial ATP production [78]. Further studies are needed to address these details in neuronal cells and immune cells and to determine whether a direct effect of delivered oxygen, an indirect effect through PI3K-Akt activation, or both are behind the increased mitochondrial ATP generation in response to RNS60.

Lastly, treatment with RNS60, an oxygen-containing drug, did not seem to cause any additional oxidative stress, and to the contrary, resulted signs of an anti-oxidant effect. Nrf2 is a basic region leucine-zipper transcription factor that binds to the antioxidant response element (ARE) and activates a battery of genes involved in the cellular antioxidant and anti-inflammatory defense, as well as mitochondrial biogenesis [79]. Reduction of Nrf2 expression levels in motor neurons and astrocytes has been demonstrated in ALS mouse models and patients [21, 79] and is thought to contribute to chronic motor neuron degeneration. In contrast, activation of Nrf2 is neuroprotective in a mouse model of ALS [80]. Thus, the prevention of Nrf2 reduction by RNS60 observed here is likely tied to an anti-oxidant response that is associated with its protective action on MNs. The reduction of NT levels seen in RNS60-treated C57BL/6-SOD1^G93A^ mice supports this conclusion.

## Conclusions

In summary, the present study demonstrated that intraperitoneal treatment with RNS60 starting from onset of the disease delays the onset of neuromuscular deficit and paralysis, and increases the survival interval from onset in C57BL/6-SOD1^G93A^ mice by about 17%. This effect seems to be similar to or better than that obtained with other treatments targeting neuroinflammation, mitochondrial dysfunction, and oxidative stress [81], including that of edaravone, an antioxidant which has been recently approved by FDA for ALS treatment [82]. Notably RNS60, like edaravone, displayed a delay in motor decline with a trend to survival extension when the treatment started at the onset of symptoms. This is important in light of the translation to clinical practice where patients can only be treated at the diagnosis, often after the symptom onset. Moreover, RNS60 has a simple chemical composition (NaCl, oxygen, and water), which reduces the likelihood of side effects caused by metabolic by-products and may allow it to be safe when used in combination with other compounds. These significant preclinical findings, together with the excellent clinical safety profile, make RNS60 a promising candidate for ALS therapy and support further studies to unravel its therapeutic potential and molecular mechanism of action.

## Additional files

Additional file 1: **Figure S1.** Primary microglia-MN enriched co-cultures exposed to LPS for 24 h after 6 DIV. The bar graph indicates that LPS reduces the viability of MNs treated with 10% (*v*/*v*) NS or ONS60 (ONS) by about 30%. The toxic effect was significantly prevented by RNS60 (10% *v*/*v*). Data are expressed as mean ± SEM (*n* = 6), One-way ANOVA (*p* < 0.001) followed by post hoc Fisher’s LSD. *** = *p* < 0.001. (DOCX 185 kb)

Additional file 2: **Figure S2.** Representative immunoblot for GFAP performed on ventral portion of LSC of NTG mice or transgenic mice treated with NS or RNS60, at 20 weeks of age, and relative quantification. Data are expressed as mean ± SEM, (*n* = 5 animals per group). Data were statistically analyzed using one way ANOVA followed by post hoc Fisher’s LSD. * = *p* < 0.05, ** = *p* < 0.01, *** = *p* < 0.001, n.s. = non significant. (DOCX 162 kb)

Additional file 3: **Figure S3.** A-D) Representative images of LSC micrographs stained with S100β (green) and GFAP (red) at 20 weeks of age. Scale bar: 20 μm. E) Quantification of immunofluorescence showed no differences between the two transgenic groups. Bar graphs represents mean ± SEM, (*n* = 5 animals per group); One-way ANOVA followed by post hoc Fisher’s LSD non-parametric test (*p* = 0.420). (DOCX 1342 kb)

## Abbreviations

ALS: Amyotrophic lateral sclerosis
ATP: Adenosine triphosphate
BSA: Bovine serum albumin
CCL2: Chemokine (C-C motif) ligand 2
CNPase: 2′,3′-Cyclic nucleotide 3′-phosphodiesterase
CNS: Central nervous system
DIV: Days in vitro
FDA: Food and Drug Administration
FoxP3: Forkhead box P3
GDNF: Glial-derived neurotrophic factor
GFAP: Glial fibrillary acid protein
GSK3β: Glycogen synthase kinase
h: Human
HBBS: Hanks’ balanced salt solution
HRP: Horse radish peroxidase
IBA1: Ionized calcium-binding adapter molecule 1
IGF1: Insulin growth factor
IL-1 β: Interleukin 1 beta
IL-4: Interleukin 4
LSC: Lumbar spinal cord
LSD: Least significant difference
MBP: Myelin basic protein
MNs: Motor neurons
NGS: Normal goat serum
NMJ: Neuromuscular junction
NS: Normal saline
NT: Nitrotyrosine
NTG: Non-transgenic
PBS: Phosphate-buffered saline
PCR: Polymerization chain reaction
PFA: Paraformaldheyde
PI3K: Phosphatidylinositol-3 kinase
PNS: Peripheral nervous system
SOD1: Superoxide dismutase 1
TAM: Tibialis anterior muscle
Treg: T regulatory (cell)
VEGF: Vascular endothelial growth factor
Ym1: Chitinase-like
