# Supplementary figures and images for: RNS60 exerts therapeutic effects in the SOD1 ALS mouse model through protective glia and peripheral nerve rescue
Source: J Neuroinflammation. 2018 Mar 1;15:65. doi: 10.1186/s12974-018-1101-0 (PMC5833072; doi:10.1186/s12974-018-1101-0)

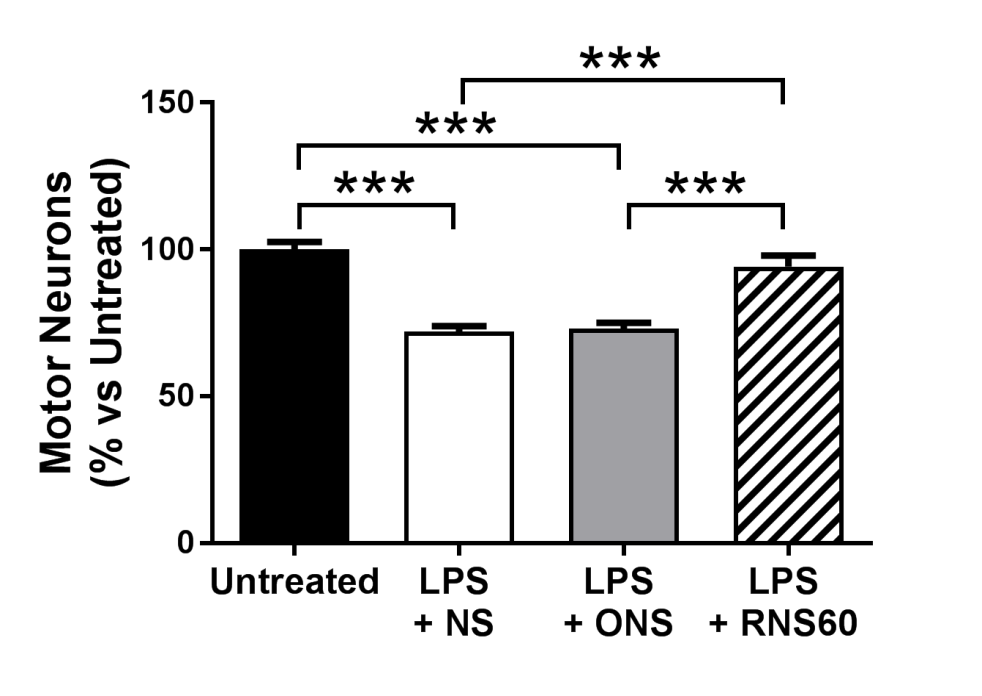

Supplement: Supplementary file 1 — Figure S1. Primary microglia-MN enriched co-cultures exposed to LPS for 24 h after 6 DIV. The bar graph indicates that LPS reduces the viability of MNs treated with 10% (v/v) NS or ONS60 (ONS) by about 30%. The toxic effect was significantly prevented by RNS60 (10% v/v). Data are expressed as mean ± SEM (n = 6), One-way ANOVA (p < 0.001) followed by post hoc Fisher’s LSD. *** = p < 0.001. (DOCX 185 kb) [file 12974_2018_1101_MOESM1_ESM.docx]

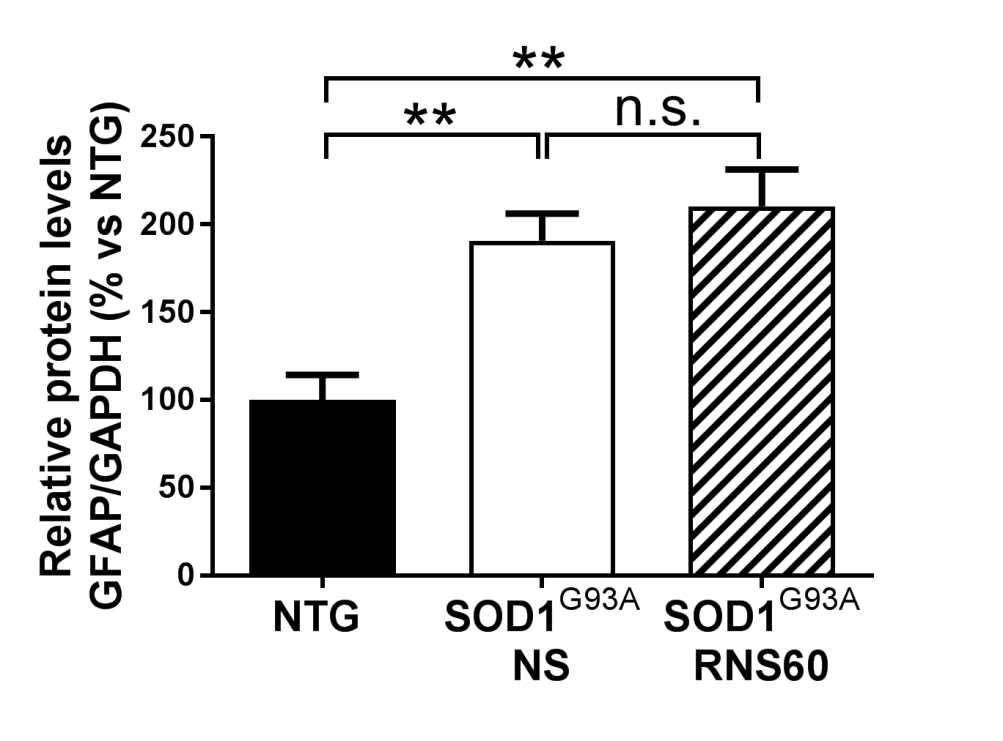

Supplement: Supplementary file 2 — Figure S2. Representative immunoblot for GFAP performed on ventral portion of LSC of NTG mice or transgenic mice treated with NS or RNS60, at 20 weeks of age, and relative quantification. Data are expressed as mean ± SEM, (n = 5 animals per group). Data were statistically analyzed using one way ANOVA followed by post hoc Fisher’s LSD. * = p < 0.05, ** = p < 0.01, *** = p < 0.001, n.s. = non significant. (DOCX 162 kb) [file 12974_2018_1101_MOESM2_ESM.docx]

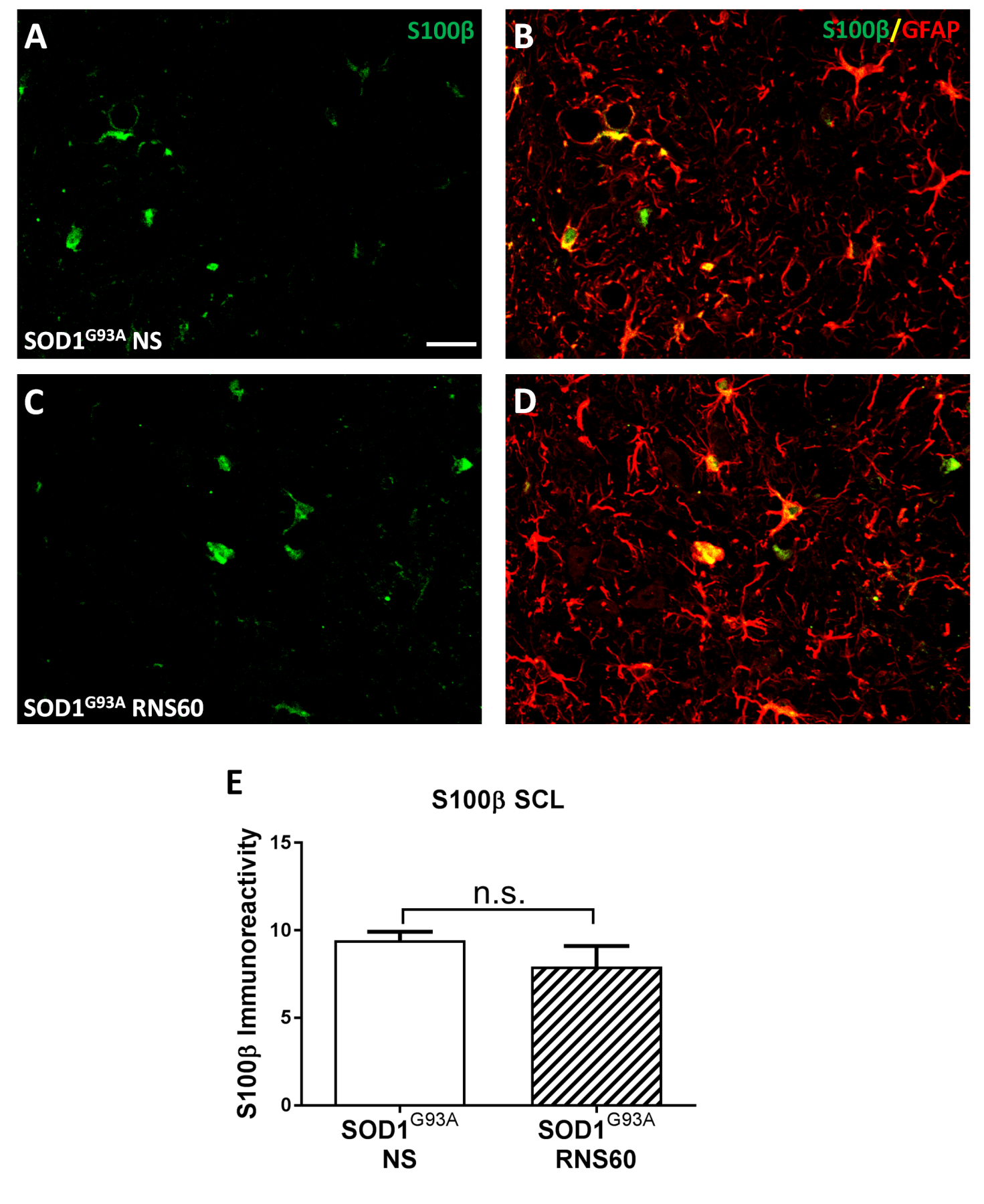

Supplement: Supplementary file 3 — Figure S3. A-D) Representative images of LSC micrographs stained with S100β (green) and GFAP (red) at 20 weeks of age. Scale bar: 20 μm. E) Quantification of immunofluorescence showed no differences between the two transgenic groups. Bar graphs represents mean ± SEM, (n = 5 animals per group); One-way ANOVA followed by post hoc Fisher’s LSD non-parametric test (p = 0.420). (DOCX 1342 kb) [file 12974_2018_1101_MOESM3_ESM.docx]
